# Electrospun Scaffolds for Osteoblast Cells: Peptide-Induced Concentration-Dependent Improvements of Polycaprolactone

**DOI:** 10.1371/journal.pone.0137505

**Published:** 2015-09-11

**Authors:** Monica Dettin, Annj Zamuner, Martina Roso, Antonio Gloria, Giovanna Iucci, Grazia M. L. Messina, Ugo D'Amora, Giovanni Marletta, Michele Modesti, Ignazio Castagliuolo, Paola Brun

**Affiliations:** 1 Department of Industrial Engineering, University of Padova, Padova, Italy; 2 Institute of Polymers, Composites and Biomaterials—National Research Council of Italy, Naples, Italy; 3 Department of Physics, University “Roma Tre”, Roma, Italy; 4 Department of Chemistry, University of Catania, Catania, Italy; 5 Department of Molecular Medicine, University of Padova, Padova, Italy; University of Milan-Bicocca, ITALY

## Abstract

The design of hybrid poly-ε-caprolactone (PCL)-self-assembling peptides (SAPs) matrices represents a simple method for the surface functionalization of synthetic scaffolds, which is essential for cell compatibility. This study investigates the influence of increasing concentrations (2.5%, 5%, 10% and 15% w/w SAP compared to PCL) of three different SAPs on the physico-chemical/mechanical and biological properties of PCL fibers. We demonstrated that physico-chemical surface characteristics were slightly improved at increasing SAP concentrations: the fiber diameter increased; surface wettability increased with the first SAP addition (2.5%) and slightly less for the following ones; SAP-surface density increased but no change in the conformation was registered. These results could allow engineering matrices with structural characteristics and desired wettability according to the needs and the cell system used. The biological and mechanical characteristics of these scaffolds showed a particular trend at increasing SAP concentrations suggesting a prevailing correlation between cell behavior and mechanical features of the matrices. As compared with bare PCL, SAP enrichment increased the number of metabolic active h-osteoblast cells, fostered the expression of specific osteoblast-related mRNA transcripts, and guided calcium deposition, revealing the potential application of PCL-SAP scaffolds for the maintenance of osteoblast phenotype.

## Introduction

Most of the synthetic polymers used used in engineering of cells are characterized by good mechanical and physical properties but are usually hydrophobic and often lack suitable biocompatible molecular sites for communicating with target cells. For example, poly- ε-caprolactone (PCL) has been widely used in several biomedical applications due to its biodegradability, biocompatibility, and good mechanical properties. PCL cast films however are not appropriate for cell scaffolding because they are non-porous and therefore prevent nutrients and oxygen from being transported to the cells.

In the last decade, electrospinning has emerged as a new technique able to weave fibers that are structurally similar to the fibrous components of most extracellular matrices (ECMs). Fiber composition, diameter, alignment and scaffold porosity can be tailored to the particular cell or tissue type [1, 2]. In addition, the high area-to-volume ratio offers the possibility to improve surface decoration with protein, peptides or other bioactive molecules.

In order to modify synthetic electrospun scaffolds for obtaining increased hydrophilicity and additional biomolecules, several techniques have been proposed such as plasma modification, wet chemical method, surface graft polymerization, blending of synthetic polymers with natural ones, or physical adsorption of proteins [3–7].

The covalent grafting of bioactive molecules on the scaffold surface has the advantage of not allowing molecules to leach from the surface.

However, this technique does not account for several other key (and onerous) requirements in the design of last generation bioactive surfaces, such as orientation, concentration, distribution of the biomolecules on the surface, preservation of the native structure and of the side chain groups in surface chemistry, which are required for recognition of cell receptors and subsequent cell binding.

Hydrogels custom-made using physiological fibril-SAPs are chemically defined extracellular matrices capable of sustaining tissue regeneration on their own or by following functionalization with biomolecules such as cytokines or growth factors that could be included in the nanofibrous material or covalently attached to it [8, 9]. These new biomaterials based on synthetic peptides are composed of self-complementary sequences in which amino acids with a positive charge and residues with a negative charge are separated by hydrophobic residues. According to the proposed model [10], membranes are formed by the stacking of *β-sheets* layers; each one binds to the preceding one by ionic bridges formed between charged groups of lateral chains, and to the subsequent sheet via hydrophobic interactions.

Interestingly, scaffolds made of SAPs support adhesion and proliferation of differentiated cells and also stimulate cell differentiation of immature stem cells [11]. However, since SAP hydrogels are characterized by a relatively low amount of moduli (up to about 10 kPa), they have mainly been used as injectable scaffolds to treat small defects rather than patches for large ones. In an effort to optimize surface biocompatibility and stiffness of ECM-mimetic electrospun fiber scaffolds, we blended a biodegradable FDA-approved polymer (PCL) with self-assembling peptides [12]. Our aims were to: i) correct the high hydrophobicity of PCL and lack of biochemical signals; ii) rectify the excessive softness of peptide hydrogels. Actually, we previously reported that enrichment of PCL with 5% of SAPs increased surface wettability and enhanced h-osteoblast adhesion, differentiation and expression of hALP, hOPN and hBSP genes [12].

In this study, the three self-assembling peptides (Table 1), which reported the best results in biological assays [12], were exploited to produce hybrid matrices with increasing peptide concentrations. The aim was to assess the physico-chemical, mechanical and biological modifications induced by increasing SAP concentrations. The hybrid matrices were characterized based on the diameter of the fibers, the wettability, the surface concentration of N-containing groups and the mechanical properties. In addition, FT-IR measurements revealed the secondary structure of the incorporated peptides. In actual fact, the enrichment of PCL with increasing concentrations of peptides causes the features of the scaffold to modulate. Thus, it should be noted that the percentage of peptides embedded in the scaffolds is directly correlated to the diameter of the fibers. Furthermore, hydrophilicity of the matrix surfaces increased with the increase in the percentage of peptides in the PCL solution before electrospinning. The enrichment of PCL with SAPs produces different performances in tensile modulus, maximum stress and maximum strain that vary from the sequence chosen. As demonstrated by biological assays, matrices characterized by a specific peptide percentage for each SAP (10% for RGD-EAK; 5% for EAbuK and 15% for EAK) promote h-osteoblasts vitality, support calcium deposition and increase osteoblast-related genes expression.

**Table 1:** The names and sequences of the synthetic SAPs.

Name	Sequence
EAK	AEAEAKAKAEAEAKAK
EAbuK	AbuEAbuEAbuKAbuKAbuEAbuEAbuKAbuK
RGD-EAK	RGDAEAEAKAKAEAEAKAK

All the SAPs were synthesized as C-terminal amides. Abu refers to α-aminobutyric acid.

## Materials and Methods

### Materials

The solid support (resin Rink-Amide MBHA) and the Fmoc protected amino acids were from Novabiochem (Merck KGaA, Darmstadt, Germany). The coupling reagents 2-(1H –Benzotriazole-1-yl) -1,1,3,3-tetramethyluronium hexafluorophosphate (HBTU) and 1-Hydroxybenzotriazole (HOBt) were from Advanced Biotech (Seveso, MI, Italy). N,N-diisopropylethylamine (DIEA) and piperidine were from Biosolve (Leenderweg, Valkenswaard, Netherlands). Triethoxysilane (TES), were from Sigma-Aldrich (Steinheim, Germany). Solvents such as N,N-dimethylformamide (DMF), trifluoroacetic acid (TFA), N-methyl-2-pyrrolidone (NMP) and dichloromethane (DCM) were from Biosolve (Leenderweg, Valkenswaard, Netherlands). The solvent for electrospinning solutions hexafluoroisopropanol (HFIP) was from Alfa Aesar (Ward Hill, MA, USA). Poly-ε-caprolactone, average M_n_ ca. 60,000 (PCL) used to produce the electrospun scaffolds was from Aldrich (St. Louis, MO, USA).

### Peptide synthesis

#### EAK

The self-assembling peptide EAK (Table 1) was synthesized and purified as reported in ref. [13].

#### RGD-EAK

This peptide presents the adhesion motif Arg-Gly-Asp (RGD) condensed to the EAK sequence (Table 1). The details of its synthesis are reported in S1 Protocol.

#### EAbuK

The synthesis of EAbuK (Table 1) is reported in S1 Protocol.

#### GE3M

This peptide (sequence: H-Ile-His-Ile-Gly-Pro-Gly-Arg-Ala-Phe-Tyr-Thr-Thr-OH) was synthesized and purified as reported in reference [14]. The peptide GE3M has not the typical pattern of SAPs and so it is unable to aggregate. GE3M was chosen as negative control because its M_W_ and length are similar to SAPs' ones.

### Preparation of electrospun scaffolds

#### Solutions for electrospinning

All solutions were prepared dissolving 13% (wt/wt) of solute in HFIP. For example, the polymer/peptide solution used to produce 2.5% RGD-EAK scaffold was prepared by dissolving 1.79 mg of RGD-EAK in 0.3 mL HFIP and successively adding 69.32 mg of PCL. The exact quantity of peptides and PCL used are reported in S1 Table. A control solution (13% wt/wt) containing only PCL was also prepared.

#### Air blowing assisted electrospinning

During the electrospinning process, the solution was released from a 2.5 mL syringe through a 27G needle at a 0.2 mL/h flow rate by a volumetric pump; the applied voltage between the electrodes was 20 kV, and the air flow pressure was 0.2 bar. Fibers were collected for 1.5 h on an aluminium foil (7×8 cm) at a distance of 22 cm from the needle. After deposition all the samples were dried under vacuum for 1 h in the presence of P_2_O_5_. The relative humidity (RH%) and temperature (°C) were monitored during the electrospinning process and kept at a 60% RH and 24±0.5°C.

### Scanning electron microscopy

Electrospun scaffolds were sputter coated with gold (EMITECHK950x Turbo Evaporator, EBSciences, East Granby, CT) and observed by means of Scanning Electron Microscopy (SEM; Cambridge Stereoscan 440 SEM, Cambridge, UK). Images were acquired at 500x, 5,000x and 30,000x magnifications with an accelerating voltage of 20 kV. The diameter range of the fabricated nanofibers was measured using commercial imaging software (ImageJ, National Institutes of Health, Bethesda, MD). Three representative images, in three different areas, were chosen for every sample.

### Statistical analysis of process parameters

The screening experiments performed in the previous work [12] and the OFAT analysis (One Factor at one Time) allowed the investigations to set the proper range of operating conditions in terms of applied voltage, flow rate, needle-collector distance, and ambient parameters, in order to obtain smooth PCL nanofibers with a lower content of bead-defects.

In this study we evaluated the impact of the concentrations of the different peptides on the diameter of the fibers. Thus, being all the other conditions set as aforementioned in the *Preparation of electrospun scaffolds* section, the mean of fiber diameters (y) was analyzed in accordance to the following factors:

A: peptide concentration (numeric factor);
B: sequence type (categorical nominal factor)

The parameters and the relative data are summarized in S2 Table.

### Contact angle measurements

The surface wettability was traced by measuring the static water contact angle. An OCA30 instrument (Dataphysics), equipped with a CCD camera for the drop shape analysis, was used at 25°C and 65% relative humidity. The scaffolds electrospun on aluminum foil were fixed on glass coverslips by double-sided tape. Two μL of ultrapure water were applied on different areas of the surface. The static contact angles were then measured on both sides of the two-dimensional projection of the droplet by digital image analysis. Data are reported as the average of at least three separate measurements.

### XPS measurements

XPS studies were performed using a homemade instrument with preparation and analysis chambers separated by a gate valve. The analysis chamber is equipped with a six degrees of freedom manipulator and a 150 mm mean radius hemispherical electron analyzer with five-lens output system combined with a 16-channel detector. Measurements were performed on at least two different specimens for each scaffold in order to check data reproducibility; average values are reported. C1s, O1s and N1s core level signals were recorded.

The measured Binding Energies BE (±0.1 eV) were calibrated to the C1s signal of aliphatic-aromatic C-C carbons located at a BE = 285.0 eV [15, 16]. Experimental spectra were analyzed by curve fitting using Gaussian curves as fitting functions. The C1s spectra were fitted using three component peaks labelled C_1_, C_2_, C_3_ at increasing BE order, corresponding to aliphatic C-C carbons (C_1_ BE = 285.0 eV), to C-O carbons (C_2_ BE = 286.5 eV), and to carboxyl O-C = O carbons (C_3_ BE = 288.8 eV), by comparison with literature data [15]. In the reported experimental conditions, we could not resolve by fitting the C_3_ signals related to carboxylic carbons of PCL and to peptide carbons (BE = 288.4 eV [17, 18]), of the SAPs. The O1s signal consists of two peaks, corresponding to carbonyl O = C oxygens (O_1_ BE = 531.9 eV) and to O-C oxygens (O_2_ BE = 533.4 eV) [15]. The N1s signal appears symmetrical and therefore was fitted with a single peak located at a BE in the range 399.8–399.9 eV typical of amide nitrogens [17, 18]. Atomic ratios (±10%) were calculated from peaks areas using Scofield’s cross section as sensitivity factors.

### FT-IR spectroscopy

Reflection absorption infrared spectra (RAIRS) of the polymer nanofibres deposited onto aluminum foil were recorded by means of a VECTOR 22 (Bruker, Billerica, MA) FT-IR interferometer, equipped with a DTGS detector and with a reflectance/grazing angle accessory (Specac, Orpington, UK). The incidence angle of the impinging radiation was 70°. Measurements were taken at least on two different specimens for each scaffold.

### Tensile Tests

Electrospun PCL-based meshes were immersed at 37°C in physiological solution. Tensile tests were carried out on wet specimens with a total length of 20 mm, a thickness (t) and a width (w) which fall in the range of 0.01÷0.07 mm and 6.0 ÷ 8.0 mm, respectively. The grip-to-grip distance (l_0_) (i.e. gauge length) was 10 mm. All the tests were performed on wet specimens at a rate of 10 mm/min using an INSTRON 5566 testing machine. The engineering stress (σ) was calculated as follows: *σ = F/A*; where *F* represents the force measured by the load cell and A is the cross section (t w).The engineering strain (ε) was evaluated as the ratio between the elongation *Δl* and the gauge length *l*_*0*_: *ε = Δl / l*_*0*._ Tensile modulus, maximum stress and maximum strain were evaluated. The results were analyzed using the analysis of variance (ANOVA) followed by Bonferroni post-hoc tests. Statistical differences were set at *p* < 0.05.

### Biological Characterization

#### Cell culture

Electrospun scaffolds were cut from each aluminum foil into disks with diameter of 1.4 cm and secured into 24-well tissue culture plates (Costar, Turin, Italy). To avoid bacterial contamination, scaffolds were incubated in ethanol 20% for 10 min and then extensively washed in sterile phosphate buffered saline (PBS). Human (h) osteoblast cells were obtained from explants of cortical mandible bone collected during a surgical procedure from a healthy 66 year-old male subject. The study was approved by the Ethical Committee of the University Hospital of Padova. The patient was informed of the study aims and protocol and gave his written informed consent. Bone fragments were cultured in complete medium (composition reported in S2 Protocol) and incubated at 37°C until cells migrated from the bone fragments. At cell confluence, bone fragments were removed. Cells were detached using trypsin–EDTA (Gibco) and cultured in complete medium supplemented with 50 μg/mL ascorbic acid, 10nM dexametasone, and 10mM β-glycerophosphate (all from Sigma-Aldrich). At the 10th day of culture osteoblast phenotype was confirmed by the von Kossa staining [19]. In this study h-osteoblasts were used at passage 5th–8th in culture. Cells (1.3×10^5^ cells/cm^2^) were seeded onto electrospun matrices in 100 μL of complete culture medium. Cultures were then incubated for different time extent as specified for each experiment in a humidified tissue culture incubator (Heraeus; Corston, Bath, UK) at 37°C in 5% CO_2_ and 95% humidity. The incubator was also equipped with an additional pan of sterile water to prevent evaporation of tissue culture media. The volume and the pH of the complete medium were checked every 24 h.

#### Cell vitality assay

Cellular vitality was assessed by using the MTT (3-(4,5-dimethylthiazole-2-yl)-2,5-diphenyl tetrazoliumbromide) assay as reported in S2 Protocol. H-osteoblasts seeded on electrospun matrices were incubated at 37°C for 2 h, incubation time previously reported ensuring the optimal adhesion of h-osteoblasts to functionalized surfaces [20].

#### Quantitative real time polymerase chain reaction

Human osteonectin (SPARC), integrin-binding sialoprotein (IBSP), and runt-related transcription factor 2 (RUNX2), and marker of proliferation Ki-67 (MKI67)mRNA specific transcript levels were quantified in osteoblast cells cultured for 24 h on different electrospun scaffolds. At the end of incubation, total RNA was obtained using the SV Total RNA Isolation System kit (Promega, Milan, Italy). Contaminating DNA was removed by DNase I digestion and 1 μg of total RNA were retrotranscribed to cDNAs using Moloney Murine Leukemia Virus reverse transcriptase (Applied Biosystems, Milan, Italy). Quantitative PCR was performed using the ABI PRISM 7700 Sequence Detection System (Applied Biosystems, Monza, Italy), TaqManqPCR Master Mix (Applied Biosystems), primers designed using Primer Express software, and probes from the Universal Probe Library system (UPL, Roche, Applied Science, Monza, Italy). The melting temperature was 60°C. Data were quantified by the ΔΔC_T_ method using hGAPDH as reference gene. Target and reference genes were amplified with efficiencies near 100%. Oligonucleotides and probes used for PCR are listed in S3 Table.

#### Calcium assay

A hallmark of osteoblast differentiation and proliferation is the formation and deposition of calcium phosphate crystals [21]. As previously reported calcium content is undetectable at 2 days of culture, peaks at 7 days, and decreases at 14 days [13]. Therefore in this study, h-osteoblasts were cultured on different electrospun matrices for 7 days as reported in S3 Protocol.

#### Statistical analysis

Biological assays were performed three times and assessed in duplicate or triplicate. Statistical analysis was performed using the one-way ANOVA test followed by Bonferroni's multi-comparison test with a minimum confidence level of 0.05 for statistical significance. Data are reported as mean±standard error of the mean.

## Results

### Scanning Electron Microscopy

The matrices containing peptides showed lower defectivity compared to PCL. The lowest defectivity was observed in matrices with the highest peptide enrichments (15% wt/wt). However, there was no difference between SAP and the control peptide (GE3M). The lower defectivity is probably due to an increase in conductivity of the solution, as all the employed peptides possess charged amino acids.

### Statistical analysis of variables

**Fig 1:**
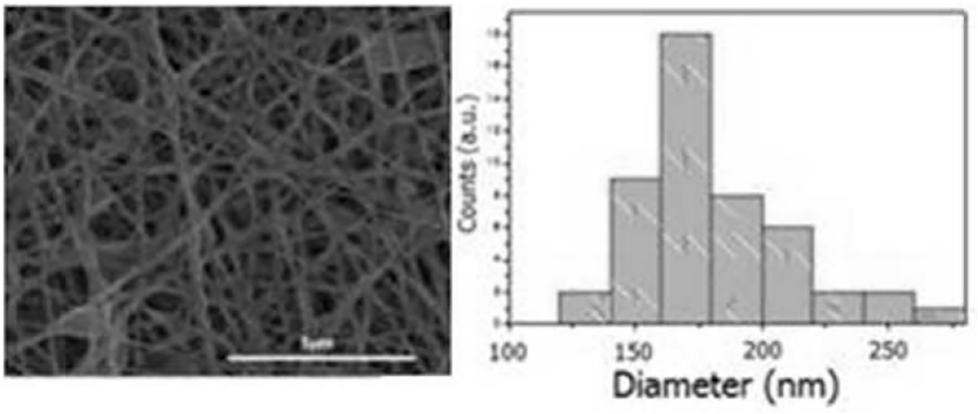
SEM Micrograph of the electrospun sample PCL-RGD-EAK 10%(wt), (a), and its diameters distribution, (b).The fiber diameter is ranging from 100 to 280 nm.

**Fig 2:**
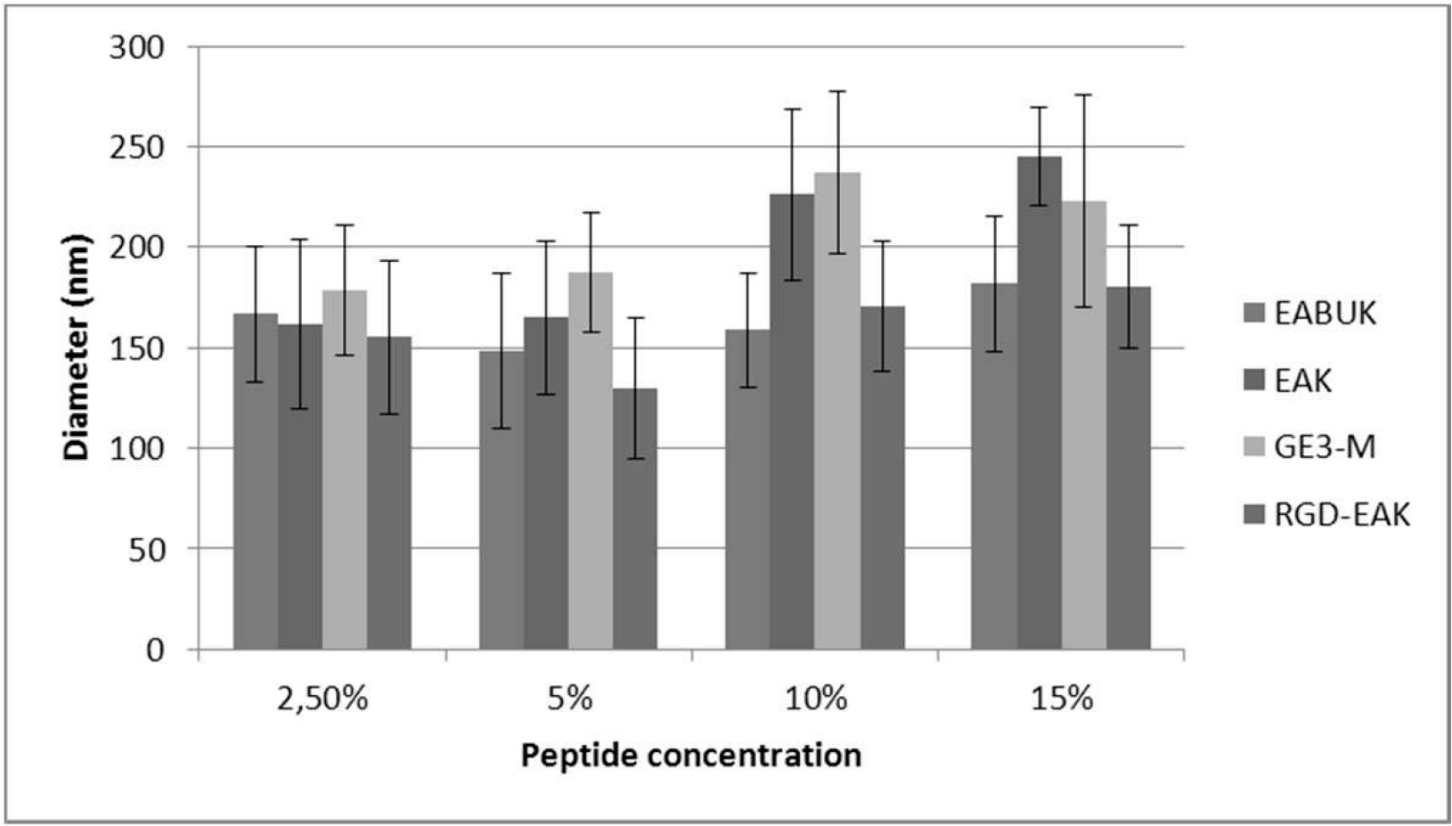
Mean fiber diameter for the tested samples.

The experiment, within the macro-area of factorial designs, was based on 16 experiments that have been summarized in S4 Table, which also shows the mean fiber diameter and standard deviation. The latter refers to the width of the distribution curve of the diameters measured as observed in Fig 1, which shows a SEM micrograph and its relative frequency distribution. However, despite the high standard deviation for each sample, the diameter of the fibers range from 100 to 280 nm. Fig 2 summarizes the mean fiber diameters of all the electrospun samples.

#### Factorial model D-optimal

The ratio between the factorial concentration and the response, and the mean diameter of fibers, was evaluated by a the following D-optimal factorial model: Y = β**0** + β**1**A + β**2**B+ β**3**C + β**12**AB + β**13**AC + …;where Yis the response, β_**n**_ is the coefficient relating to the n factor, and the letters, A, B, C, … represent the factors of the model. This model was chosen because of the historical data and the involved factors. The proposed model, in factorial form, is affected by a certain degree of accuracy and consequently is unable to explore the entire space of the independent variables, but only the fraction of space around the range of the factors. Based on the Analysis of Variance (ANOVA) applied to the 2FI model of the response (Table 2), the following statistical tests were performed:

- Test for significance of the regression model. The p-value (p < 0.0001) is less than 0.05 so it indicates that the model is significant at α = 0.05;
- Test for significance of model coefficients. The ANOVA table shows significant p-values (α = 0.05) for the main effects (α = 0.05) for each factor A: Concentration (p = 0.0001), B: Sequence (p = 0.0007), and for their interaction AB (p = 0.0353).

**Table 2:** ANOVA.

Source	Sum of Squares	df	Mean Square	F Value	p-value Prob > F
Model	19794.15	7	2827.74	16.47	0.0004
*A-Concentration*	*8346*.*66*	*1*	*8346*.*66*	*48*.*62*	*0*.*0001*
*B-Sequence*	*9017*.*69*	*3*	*3005*.*90*	*17*.*51*	*0*.*0007*
*AB*	*2429*.*80*	*3*	*809*.*93*	*4*.*72*	*0*.*0353*
Residual	1373.29	8	171.66		

Regression diagnostics were carried out to check the model’s adequacy:

- Normal Probability Plot of residuals is usefulfor checking the assumption that the errors are distributed normally. A check on the plot in S1 Fig reveals that the residuals follow a straight line and so the errors seem to be distributed normally. It should be clarified that, in this case, too many parameters are estimated to justify this test. We can consider it as a descriptive approach.
- Coefficient of determination (R^2^) measures the amount of reduction in the variability of response obtained by using all the regression variables in the model. For the specimen lifecycle model it was R^2^ = 0.9351 which means that the variation in the fiber diameter is explained reasonably well by RS approximation.

The final equations in actual terms of the fitting model for the experimental data are the following:

Sequence EAK: *DIAMETER =* 138*+*7.5 × *Concentration*

Sequence EAbuK: *DIAMETER =* 151.9*+*1.49 × *Concentration*

Sequence RGD-EAK: *DIAMETER =* 134.2 *+*3.0 × *Concentration*

Sequence GE3M: *DIAMETER =* 158.5*+*7.0 × *Concentration*

**Fig 3:**
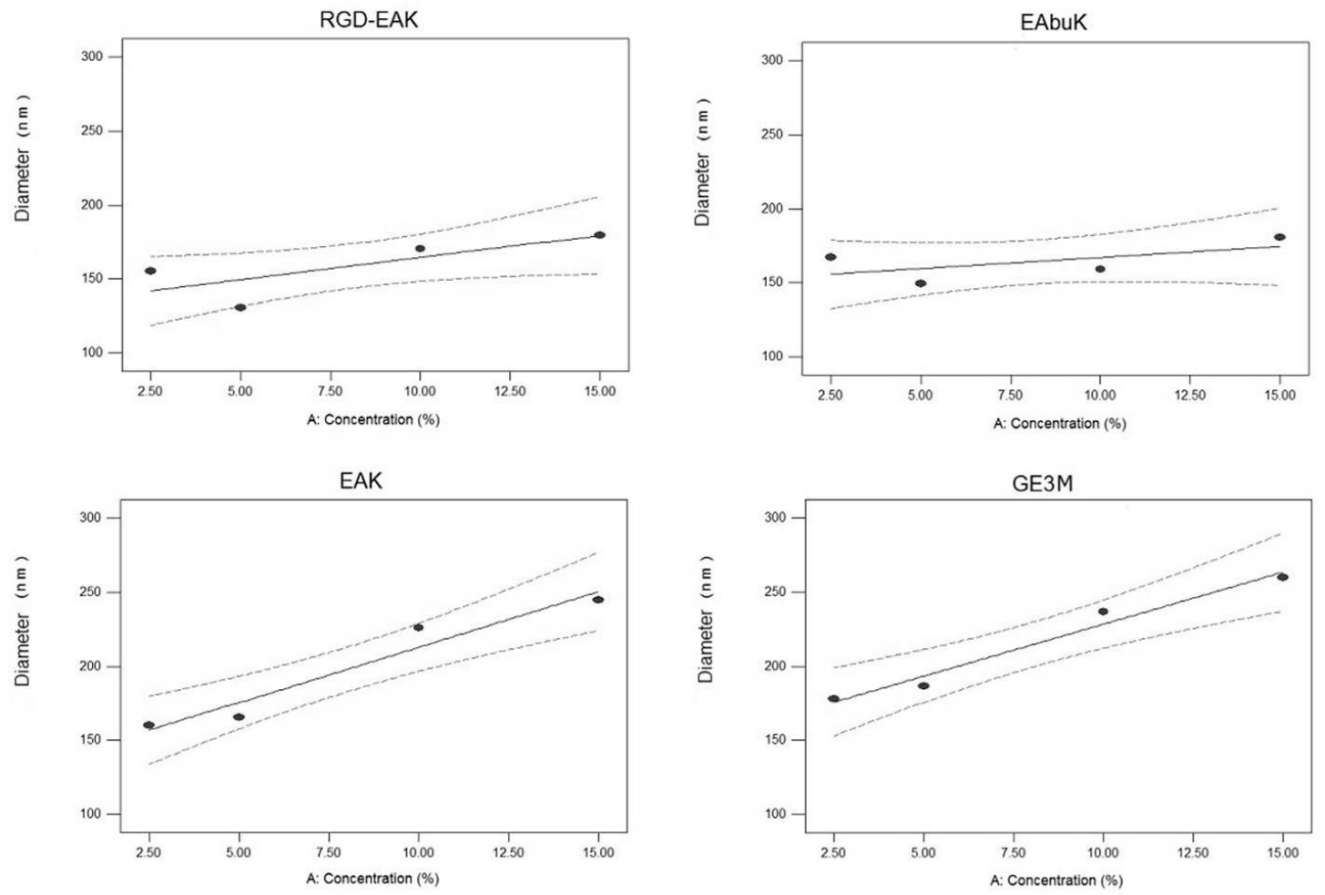
Diameter versus peptide percentage in the scaffold: experimental data (red) and fitting model. Increasing peptide concentration enhances the mean fiber diameter.

The parity plot of predicted values of the diameter compared to the actual values is shown in S2 Fig, which depicts proper model adequacy. Looking at the single peptide sequences that have been tested, it can be observed (Fig 3) that an increase in peptide concentration leads to an increase in the mean diameter of the fibers, especially in samples enriched with EAK and GE3M sequence.

This behavior can be related to the variation of the solution properties, by means that both the electrical conductivity and viscosity of the solutions are affected by the addition of peptides. An increase in electrical conductivity usually results in a decrease of the mean fiber diameter due to a growth in the bending phenomena [22]. Nevertheless, in all high concentrations, an increase in the mean fiber diameter relates more to the increase in viscosity of the solution, which reflects a lower stretching of the electrified jet, with all other conditions being equal [23].

### Contact angle measurements

**Fig 4:**
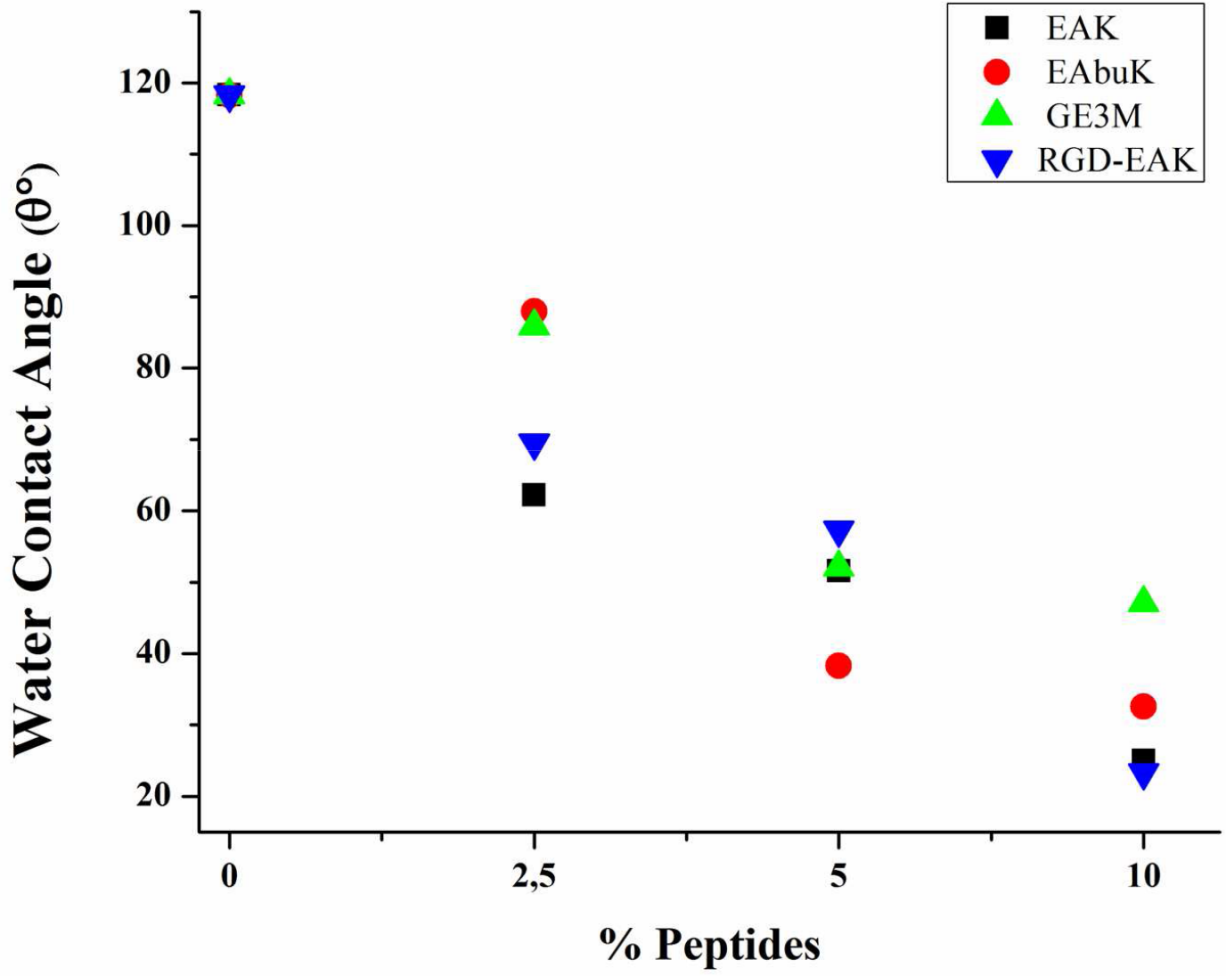
Trends of variation of *ϴ*_*c*_ for increasing concentration of different peptides. The apparent water contact angle decreases with the weight percentage increase of the added peptide from about 90° to about 25°, with a general saturation effect for the highest enrichment.

Fig 4 shows the value observed for the apparent water contact angle *ϴ*_*c*_ as a function of the increasing content of the four different peptides investigated here: EAK, EAbuK, EAK-RGD and GE3M. A significant downward trend of *ϴ*_*c*_ values for increasing peptide weight percentage is observed in all samples. Indeed, the apparent water contact angle *ϴ*_*c*_ decreases with the increase (weight percentage) of added peptide, from about 90° to about 25°, with a general saturation at the lowest *ϴ*_*c*_ values around 25° for the highest weight % of added peptides. In order to understand the nature of the apparent contact angle decrease for increasing peptide content, we have analyzed in detail the physical factors determining *ϴ*_*c*_. In a first approximation, the measured apparent water contact angle *ϴ*_*c*_ must be considered an “effective” physical quantity, describing the combined effect of the surface free energy and the complex morphology of the samples, consisting of a randomly interconnected network of sub-micron fibers. The apparent contact angle for heterogeneous surfaces is generally rationalized under the Cassie-Baxter model, which for porous samples requires the size ratio of the sessile water droplet (around 1 mm in radius) compared to the estimated pore size (<5 μm) to be very large [24, 25]. The *θ*_*c*_ values can therefore be analyzed by means of a general Cassie-Baxter equation [26]: *cosϴ*_*c*_ *= p cosϴ*_*flat*_ *—*(1-*p*), (1); where *ϴ*_*c*_ is the “apparent” contact angle, *ϴ*_*flat*_ is the true contact angle for the fiber considered a flat surface, but where *p* describes the “porosity”. In our case, the calculation of the “normalization” factor for all the samples provided is generally higher than 1, suggesting that this factor does not represent porosity alone, which by definition must be confined between 0 (for atomically flat and completely wet surfaces) and 1 for an ideal drop on air. Thus, considering the fact that the SEM analysis has shown that the dimensions of the mesh of the interconnected fiber network are remarkably similar for the various samples, we attribute the resulting drastic increase in hydrophilicity to the enrichment of polar peptides at the fiber surface, in agreement with the XPS results reported below. The determination of surface wettability for bare electrospun PCL samples gave a contact angle of 118.3° ± 9.1°.

### XPS Analysis

As described in the experimental section, the C1s and O1s spectra of PCL show a complex structure, due to carbon and oxygen atoms in slightly different chemical environments. Measured atomic ratios between the different type of carbons (C_2_/C_1_, C_3_/C_1_), oxygens (O_2_/O_1_), between oxygen and carbons (O/C) and related values calculated on the basis of the PCL chemical structure are shown in S5 Table. There is a quite good agreement between measured and calculated data.

**Fig 5:**
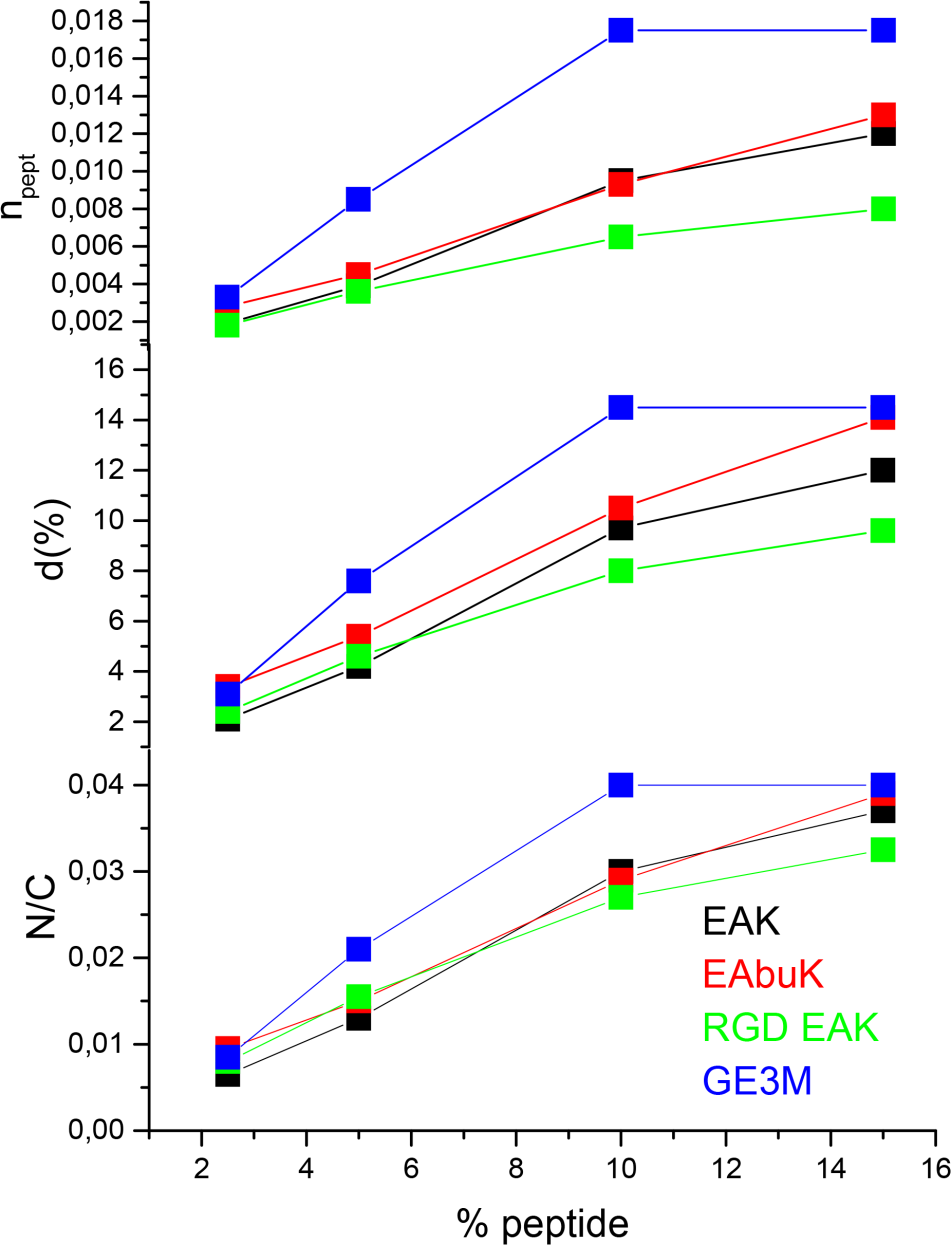
N/C atomic ratio, peptide surface density (d%) and number of peptide molecules per PCL monomer unit (n_pept_) in the investigated scaffolds versus peptide percentage in the mother solution. At increasing peptide concentration in solution, peptide surface density increases.

The C1s and O1s spectra of PCL-SAP scaffolds closely resemble the corresponding spectra recorded for pure PCL, as expected since PCL is the main component for all the investigated scaffolds. In the XPS spectra of peptides EAK, EAbuK and RGD-EAK [27–29], the C1s signal consists of three peaks, corresponding respectively to aliphatic carbons (C_1_, BE = 285.0 eV), to O = C-C-N carbons of the peptide backbone (C_2_, 286.5 eV) and to O = C-N peptidic carbons (C_3_, 288.4 eV). For all the investigated samples a new N1s peak is detected, clearly due to the peptides present in the scaffolds. Measured atomic ratios for PCL-SAP scaffolds are shown in S5 Table. The most important result is the increase in the nitrogen content, which follows the increase of the peptide percentage in the mother solution; this trend is observed in all the measured systems. Moreover, there is a slight decrease in the O_2_/O_1_ ratio with the increase of the SAP percentage; peptides in fact contain a higher percentage of O = C type oxygen (O_1_), due to the peptide bonds, and a lower percentage of O-C oxygen (O_2_) compared to PCL [27–29]. S3 Fig shows the evolution of the N1s signal as a function of SAP percentage for the PCL-EAK scaffold; a similar trend is observed for the other peptides. A plot of the N/C ratio versus peptide percentage in the scaffold for the four investigated systems is shown in Fig 5. The evident increase in the peptide nitrogen content as a function of peptide percentage shows a linear trend for the three SAPs; GE3M, on the other hand shows a saturation effect at a 10% concentration. Measured atomic ratios can be used to estimate the surface peptide density with two approaches as reported in S1 Text. In both cases, there is a good correspondence between measured peptide surface density and the peptide concentration in the mother solution. Fig 5 shows a plot of the peptide surface density and the number of peptide molecules per PCL monomer unit as a function of the peptide percentage in the mother solution. The graphs obtained with the two methodologies are very similar and confirm the increase in the peptide amount on the sample surface (first 5–10 surface nanometers sampled by XPS spectroscopy). At low concentrations, the trend is linear but at a high concentration (10%), a saturation effect is evidenced for GE3M. The XPS measurements for all the investigated systems confirm the successful peptide incorporation in the PCL fibers, the amount of incorporated peptide increasing with the peptide concentration in the mother solution.

### FT-IR Analysis

In the proteins and peptides IR spectra, the most diagnostic bands are those related to the peptide bonds, namely the amide I band, located between 1610 and 1690 cm^-1^, and basically related to peptide C = O stretching, and the amide II band, at about 1550 cm^-1^, related to N-H bending. The shape and position of the amide I band yields information on the peptide conformation (see more details in S2 Text). S4 Fig shows the spectrum in the 1800–1500 cm^-1^ range of the PCL-EAK scaffolds at increasing peptide concentrations. The spectra of the PCL-EAbuK and PCL-RGD-EAK scaffolds are perfectly alike. The amide I band is located approximately at the same wavenumber (1620–1630 cm^-1^) found for pure EAK and typical of β-sheet conformation [30], evidencing that the formation of the PCL-EAK scaffold does not affect the secondary structure of SAPs. The same results are obtained for PCL-EAbuK and PCL-RGD-EAK scaffolds.

### Mechanical analysis

**Fig 6:**
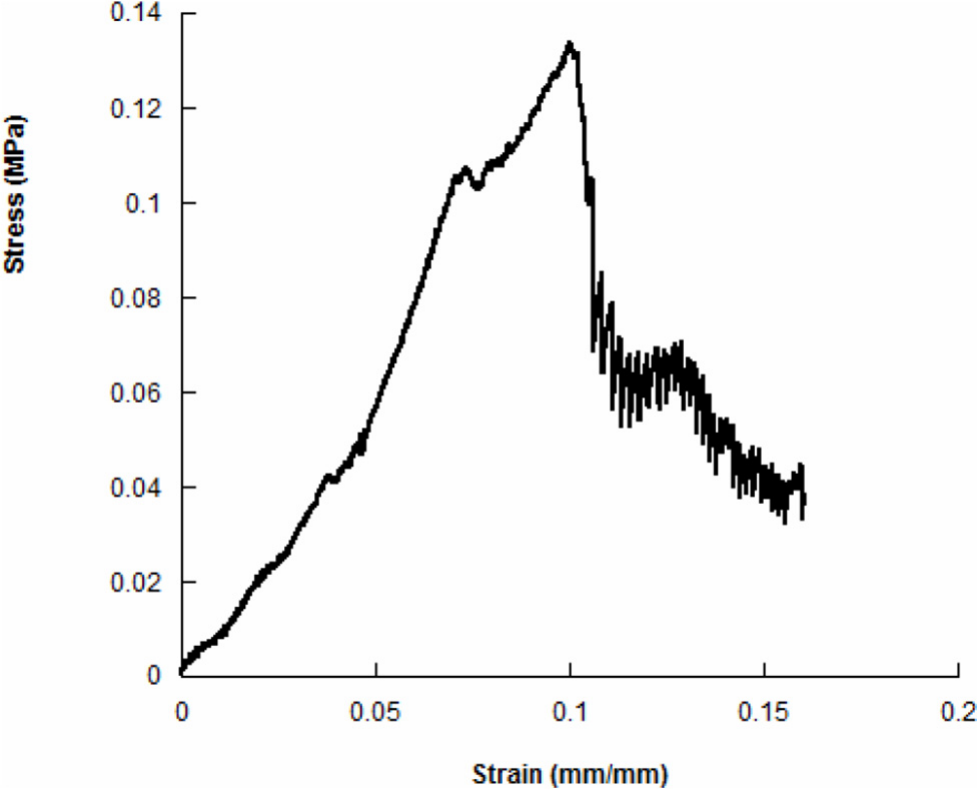
Results from tensile tests: typical stress-strain curve for the analysed electrospun meshes.

Results from tensile tests on all of the kinds of the electrospun meshes showed similar stress-strain curves, however evidencing different values of tensile modulus, maximum stress and maximum strain. A typical stress-strain curve obtained from tensile tests is reported in the Fig 6.

The stress-strain curve generally showed an initial linear region and then a change of the slope suggesting mechanical weakening and progressive local fiber failures, until a maximum stress value was reached. Successively, the first macroscopic signs of damage were evident as a consequence of fibers splitting. All the results from mechanical tests are reported in Table 3 and Table 4.

**Table 3:** Results from tensile tests on PCL electrospun meshes: modulus (E), maximum stress (σ_max_) and maximum strain (ε_max_) reported as mean value ± standard deviation.

E(MPa)	σ_max_(MPa)	ε_max_(mm/mm)
6.3±0.5	0.42±0.03	0.11±0.01

**Table 4:** Results from tensile tests on PCL-GE3M, PCL-RGD-EAK, PCL-EAbuK and PCL-EAK electrospun meshes: modulus (E), maximum stress (σ_max_) and maximum strain (ε_max_) reported as mean value ± standard deviation at different peptide concentrations. Statistical analysis was performed using analysis of variance (ANOVA) followed by Bonferroni post-hoc tests (p <0.05).

	PCL-GE3M			PCL-RGD-EAK			PCL-EAbuK			PCL-EAK		
Peptide Concentration (%)	E(MPa)	σ_max_(MPa)	ε_max_(mm/mm)	E(MPa)	σ_max_(MPa)	ε_max_(mm/mm)	E(MPa)	σ_max_(MPa)	ε_max_(mm/mm)	E(MPa)	σ_max_(MPa)	ε_max_(mm/mm)
2.5	1.2±0.1	0.25±0.03	0.20±0.01	1.0±0.1	0.05±0.01	0.09±0.01	2.7±0.2	0.21±0.02	0.07±0.01	4.1±0.5	0.24±0.03	0.08±0.01
5	3.1±0.2	0.27±0.03	0.30±0.02	5.1±0.5	0.20±0.03	0.12±0.01	4.8±0.5	0.29±0.03	0.07±0.01	1.4±0.2	0.04±0.01	0.04±0.01
10	4.1±0.2	0.28±0.03	0.36±0.02	7.2±0.5	0.50±0.03	0.14±0.01	7.8±0.8	0.38±0.03	0.08±0.01	6.3±0.5	0.13±0.01	0.16±0.02
15	5.4±0.4	0.30±0.04	0.45±0.04	9.6±0.7	1.01±0.10	0.17±0.01	2.8±0.3	0.37±0.04	0.17±0.02	1.0±0.1	0.04±0.01	0.10±0.01

Values of tensile modulus (E), maximum stress (σ_max_) and maximum strain (ε_max_) were evaluated for the neat electrospun PCL meshes (i.e., 6.3±0.5 MPa, 0.42±0.03 MPa and 0.11±0.01 mm/mm, respectively), which were basically analyzed as a control to assess the effect of the different peptides and of their concentration on the mechanical properties.

Specifically, in the case of PCL-GE3M meshes, as the GE3M peptide concentration increased from 2.5 to 15%, values of tensile modulus and maximum strain increased from 1.2±0.1 to 5.4±0.4 MPa and from 0.20±0.01 to 0.45±0.04 mm/mm, respectively. In terms of tensile modulus and maximum strain, the observed differences were statistically significant. The maximum stress was basically independent upon the peptide concentration (i.e., from 0.25±0.03 to 0.30±0.04 MPa) as statistical analysis revealed that no significant differences were found.

With regard to PCL-RGD-EAK meshes, by increasing RGD-EAK concentration from 2.5 to 15%, the modulus increased from 1.0±0.1 to 9.6±0.7 MPa. An improvement in the maximum stress (i.e., from 0.05±0.01 to 1.01±0.10 MPa) and maximum strain (i.e., from 0.09±0.01 to 0.17±0.01 mm/mm) was also evaluated. The observed differences were statistically significant.

On the other hand, in the case of PCL-EAbuK meshes the increasing peptide concentration from 2.5 to 10% enhanced the mechanical performances in terms of modulus (i.e., from 2.7±0.2 to 7.8±0.8 MPa) and maximum stress (i.e., from 0.21±0.02 to 0.38±0.03 MPa), without altering the maximum strain (i.e., from 0.07±0.01 to 0.08±0.01 mm/mm). Even though no significant strain differences were found, in terms of modulus and maximum stress the changes were statistically significant. However, by further increasing EAbuK concentration from 10 to 15% the modulus decreased from 7.8±0.8 to 2.8±0.3 MPa, the mesh flexibility increased (i.e., from 0.08±0.01 to 0.17±0.02 mm/mm) without affecting negatively the maximum stress (i.e., from 0.38±0.03 to 0.37±0.04 MPa). With regard to modulus and maximum strain the observed changes were statistically significant, whereas statistical analysis revealed no differences in the maximum stress.

A totally different effect was obtained with the EAK peptide as three different ranges of variation for the mechanical properties were found for PCL-EAK meshes. In particular, by increasing EAK concentration from 2.5 to 5.0% a decrease of tensile modulus (i.e., from 4.1±0.5 to 1.4±0.2 MPa), maximum stress (i.e., from 0.24±0.03 to 0.04±0.01 MPa) and maximum strain (i.e., from 0.08±0.01 to 0.04±0.01 mm/mm) was well evident. Anyway, as the EAK concentration further increased from 5 to 10% an improvement in tensile modulus (i.e., from 1.4±0.2 to 6.3±0.5 MPa), maximum stress (i.e., from 0.04±0.01 to 0.13±0.01 MPa) and maximum strain (i.e., from 0.04±0.01 to 0.16±0.02 mm/mm) was observed. Furthermore, in the range of 10 to 15% a new decrease of tensile modulus (i.e., from 6.3±0.5 to 1.0±0.1 MPa), maximum stress (i.e., from 0.13±0.01 to 0.04±0.01MPa) and maximum strain (i.e., from 0.16±0.02 to 0.10±0.01 mm/mm) with increasing peptide concentration was observed. Taking into account the composition ranges, with regard to the modulus, maximum stress and maximum strain, statistical analysis revealed that the observed changes were significant.

Furthermore, the obtained results showed the potential to tailor the mechanical properties of the electrospun meshes according to the specific application. As an example, it was found that even though in some cases no significant differences were found between PCL and PCL-based (i.e., PCL-EAK -10%) meshes in terms of modulus, maximum stress and maximum strain were different. In other cases, in terms of maximum stress (i.e., PCL-EAbuK -10 and 15%) or maximum strain (i.e., PCL-RGD-EAK– 2.5 and 5%) statistical analysis revealed no significant differences between PCL and PCL-based meshes. However, increasing peptide concentration, PCL-GE3M meshes also provided values of modulus (i.e., 5.4±0.4 MPa at 15%) which were close to those of PCL meshes.

### Biological results

#### Peptide concentrations influence h-osteoblast vitality

**Fig 7:**
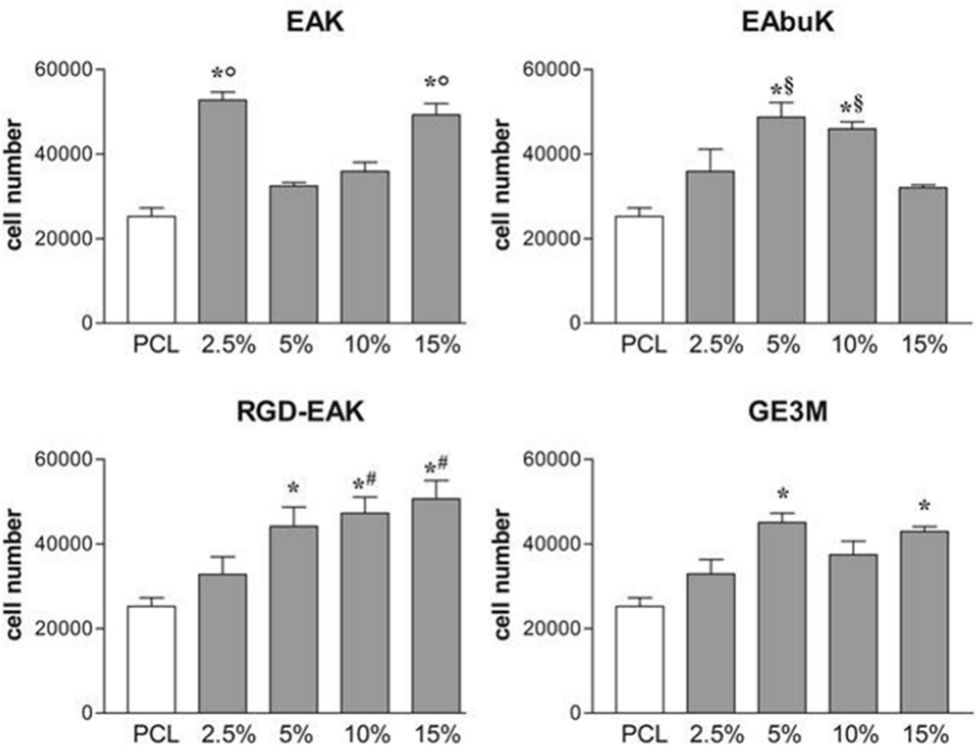
Vitality of h-osteoblast seeded for 2hon PCL-SAP matrices. All peptide enrichments (both SAP or non-SAP/GE3M) enhance osteoblast vitality *vs* PCL. EAK (2.5% and 15%), EAbuK (5% and 10%) and RGD-EAK (5%, 10% and 15%) gave the better results with respect to PCL. *denotes *P*<0.02 *vs* PCL; °denotes *P*<0.05 *vs* EAK 5% and 10%; ^§^denotes *P*<0.05 *vs* EAbuK 15%; ^#^denotes *P*<0.05 *vs* RGD-EAK 2.5%.

H-osteoblast vitality was assessed by the MTT assay. Metabolic active cells were quantified by plotting data on a standard curve as described in Materials and Methods. As reported in Fig 7, after 2 hours in culture, all the tested PCL-SAP matrices supported cell vitality as compared with PCL matrix alone although at different specific peptide concentrations. The enrichment of the PCL matrix with increasing concentrations of SAPs resulted in a progressive increment in the number of cells only when cells were cultured on RGD-EAK matrices. EAK enrichment significantly increased the cellular vitality only at concentrations of 2.5% and 15%, whereas the best performances of EAbuK were evident at concentrations of 5% and 10%, but decreased at a concentration of 15% (Fig 7). GE3M peptide also increased the number of cells as compared to the PCL matrix. However, unlike SAPs no tested concentrations of the GE3M significantly improved cell vitality compared to the other ones. As previously reported, at a 5% concentration, RGD itself increased the number of cells by more than three-fold compared with the SAP matrices [12]. The combination of RGD with EAK in the RGD-EAK SAP significantly increased cellular metabolism at concentrations of 5%, 10% and 15%, compared to PCL (Fig 7). Instead, as compared to EAK alone, the RGD-EAK matrices increased the number of cells at concentrations of 5% and 10% (S5 Fig). As reported above (Fig 4) the increased concentrations of peptides (2.5% *vs* 5% and 10%) in the RGD-EAK scaffolds increased the hydrophilicity of the surfaces resulting in improved biocompatibility. However, several other factors such as orientation, alignment, width, and dimension of the fibers of the extracellular matrix affect cell adhesion and morphology [31]. Overall, our data supported the idea that the biological activity of SAPs strictly depends on hydrophilicity and eventually on peptide concentration. Each tested peptide shows a typical range of optimal concentration as regards matrix surface features and cellular metabolism.

#### Self-assembling peptides guide osteogenic differentiation

**Fig 8:**
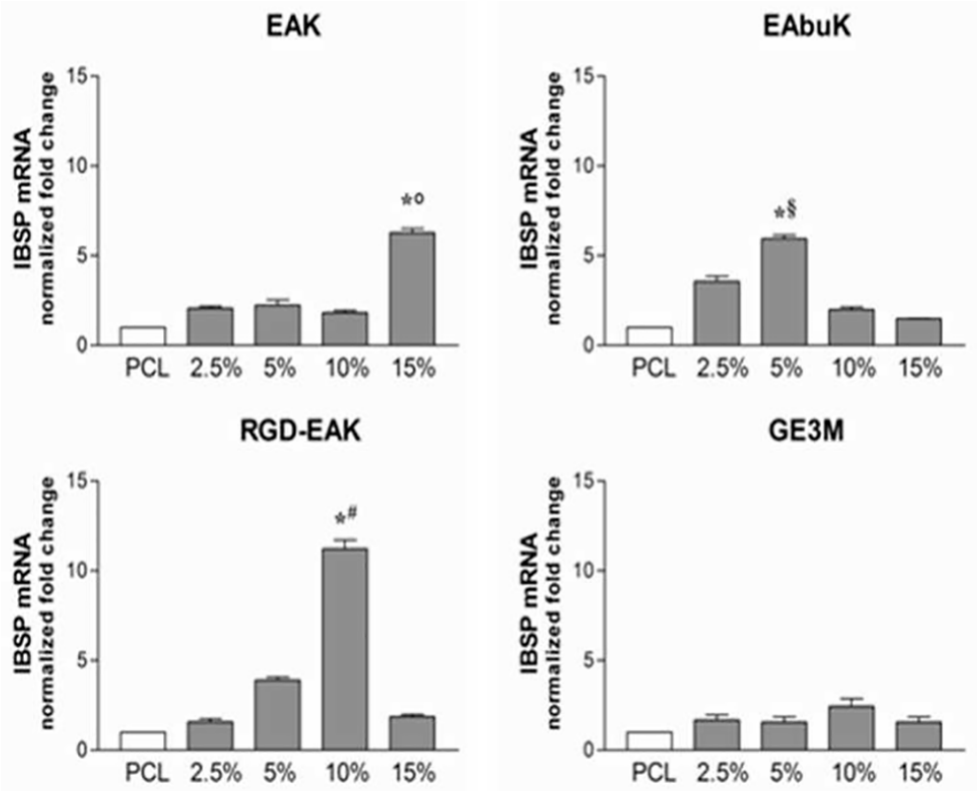
Human IBSP (integrin-binding sialoprotein) mRNA specific transcripts levels evaluated by quantitative RT-PCR. Significant increases were reported for 15%EAK, 5%EAbuK and 10% RGD-EAK whereas GE3M did not induce any improvement.*denotes *P*<0.05 *vs* PCL; °denotes *P*<0.05 *vs* EAK 2.5%, 5%, and 10%; ^§^denotes *P*<0.05 *vs* EAbuK 10% and 15%; ^#^denotes *P*<0.05 *vs* RGD-EAK 2.5%, 5%, and 15%.

**Fig 9:**
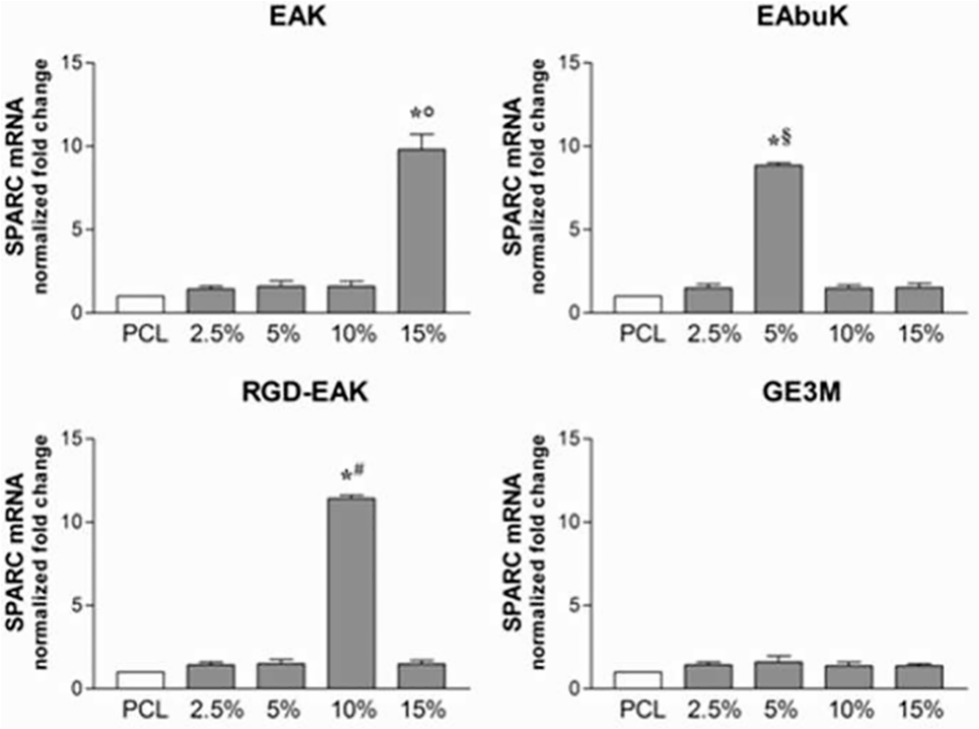
Human SPARC (osteonectin) mRNA specific transcripts levels evaluated by quantitative RT-PCR. Significant increases were reported for 15%EAK, 5%EAbuK and 10% RGD-EAK whereas GE3M did not induce any improvement.*denotes *P*<0.05 *vs* PCL; °denotes *P*<0.05 *vs* EAK 2.5%, 5%, and 10%; ^§^denotes *P*<0.05 *vs* EAbuK 2.5%, 10%, and 15%; ^#^denotes *P*<0.05 *vs* RGD-EAK 2.5%, 5%, and 15%.

**Fig 10:**
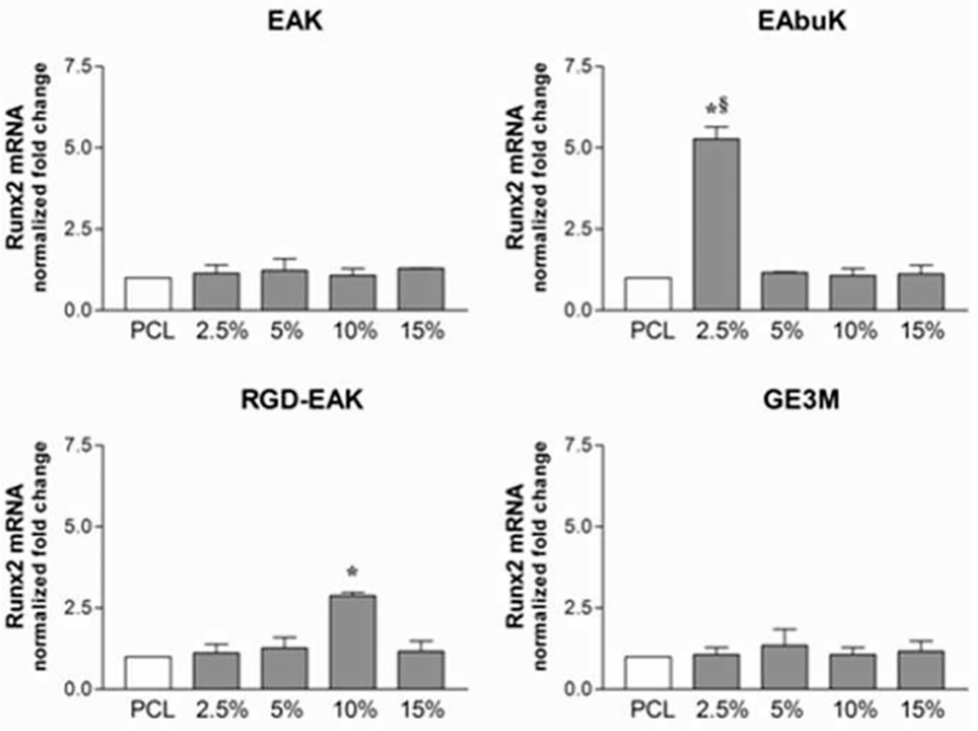
Human Runx2 (runt-related transcription factor 2) mRNA specific transcripts levels evaluated by quantitative RT-PCR. Significant increases were reported for 2.5% EAbuK and 10% RGD-EAK whereas EAK and GE3M did not induce any improvement.*denotes *P*<0.05 *vs* PCL; ^§^denotes *P*<0.05 *vs* EAbuK 5%, 10%, and 15%.

**Fig 11:**
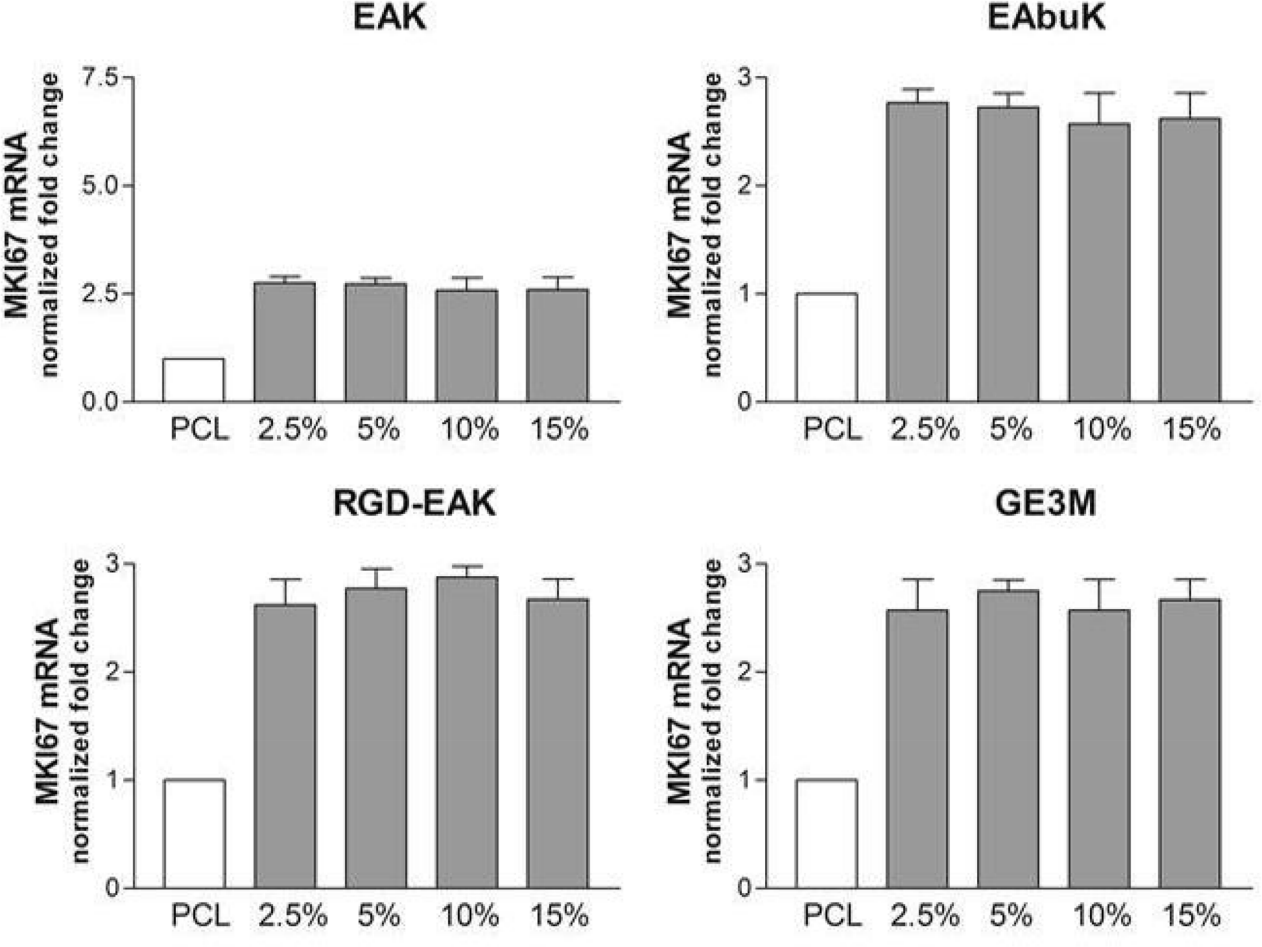
Human MKI67 (marker of proliferation Ki-67) mRNA specific transcripts levels evaluated by quantitative RT-PCR. No significant increases were reported among the different matrices.

The increased metabolic activity of h-osteoblasts seeded on the different PCL-SAP matrices only represents the first step in the osseointegration process of an implant device. Indeed, cell differentiation and mineralization of extracellular matrix determines the success of the implant [32]. To evaluate the role of different PCL-SAP matrices in sustaining h-osteoblast differentiation we quantified the mRNA transcripts coding genes specifically involved in bone formation such as IBSP, SPARC, and Runx2. As reported in Fig 8, following 24 h in culture, different concentrations of EAK (15%), EAbuK (5%), and RGD-EAK (10%) increased the levels of mRNA transcript coding IBSP, a major structural protein of the bone matrix facilitating cell attachment following recognition of RGD sequence in the extracellular matrix [33]. In fact, compared to other scaffolds, the RGD-EAK matrix mainly increasedthe expression of IBSP in h-osteoblasts. At the same peptide concentrations, EAK, EAbuK, and RGD-EAK enriched matrices also increased the levels of SPARC mRNA-specific transcripts (Fig 9). In fact, the mRNA-specific transcripts for Runx2 increased only in cells cultured on EAbuk 2.5% and RGD-EAK 10% (Fig 10). IBSP, SPARC, and Runx2 gene expressions did not increase in cells cultured on GE3M scaffold as compared to PCL. The expression of osteoblast-related genes was independent from cell proliferation or apoptosis. Thus, all the tested matrices improved cell proliferation as reported by the slight increased expression of mRNA transcripts specific for the gene MKI67 (Fig 11), marker of proliferation previously reported in proliferating osteoblasts [34].

#### Self-assembling peptides improve mineralization in h-osteoblast cultures

**Fig 12:**
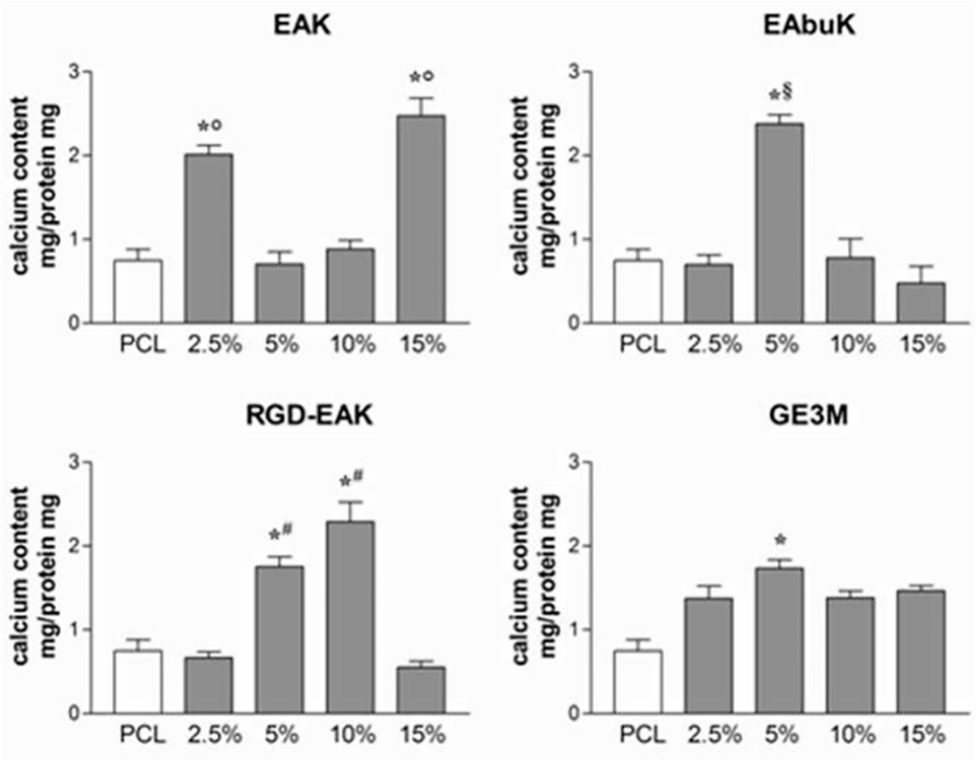
Intracellular calcium levels evaluated in h-osteoblasts cultured for 7 days onto different matrices. Both SAP and not-SAP matrices increased intracellular levels of calcium as compared to PCL.*denotes *P*<0.05 *vs* PCL; °denotes *P*<0.05 *vs* EAK 5% and 10%; ^§^denotes *P*<0.05 *vs* EAbuK 2.5%, 10%, and 15%; ^#^denotes *P*<0.05 *vs* RGD-EAK 2.5% and 15%.

The levels of calcium accumulated by cells cultured on different scaffolds for 7 days were quantified as described in Materials and Methods. As reported in Fig 12, the EAK matrix at 2.5% and 15% increased calcium depositas compared to cells cultured on PCL. EAbuk matrices induced the production of calcium only at a peptide concentration of 5%, whereas RGD-EAK increased calcium content at concentrations of 5% and 10%. In our experiments, we also observed an increased calcium content in cells cultured on a 5% peptide concentration of GE3M matrix. However, the results obtained on cells cultured on a 10% concentration of RGD-EAK scaffold were the most consistent. Thus, this scaffold promoted cell adhesion but also increased the expression of genes involved in osteoblast differentiation and extracellular matrix mineralization.

## Discussion

The electrospinning of SAPs into hybrid PCL scaffolds is a viable strategy to improve hydrophilicity and modulate cellular compatibility and degradability of biocompatible polyester matrices. This approach offers several advantages: i) easy generation of scaffolds, ii) use of physiological component (peptide) synthetically reproduced to minimize the risk of contamination; iii) covalent and selective modification of the SAPs using adhesive motives, growth factors, cross-linkers, etc. iv) improvement of the mechanical features of the matrices (i.e., from a tensile modulus of 10 kPa for pure SAPs to 9.6±0.7 MPa for PCL-RGD-EAK 15% electrospun scaffold); v) possibility to modulate the mechanical properties of the scaffolds by changing the synthetic polymer nature in the hybrid matrix; vi) the addition of a microfibrous non-woven structure as well as a nano-fibrous structure of the SAPs.

The idea of adding to the PCL a percentage of biomolecules, or the reverse, was evaluated by other authors. In particular, Heydarkhan-Hagvall S. *et al*. [35] showed that the inclusion of PCL in electrospun collagen/elastin or gelatin provides matrices that have mechanical and biological properties better than the scaffolds only made with collagen/elastin, or gelatin whose structure must be strengthened by means of crosslinking with glutaraldehyde. In fact, if the crosslinking assures good mechanical properties to the electrospun matrices, it also determines the drastic reduction of the porosity which penalizes cell behavior. In this study the authors compared the hybrid matrices varying the concentration of PCL, whereas in our study we reported the effect of peptide progressive increase in the PCL matrix. In the work of Zhang YZ *et al*. [36] the biological superiority of hybrid PCL / collagen scaffold vs PCL scaffold was proved going to compare different methods of coating (core-shell fibers vs collagen adsorbed on PCL electrospun fibers). In the work of Tambralli A. *et al*. [37] the authors used self-assembling peptides, different from ionic complementary sequences here employed, with which they functionalized by adsorption electrospun fibers of PCL: the attachment of cells was favored by the coating of the peptides. In our opinion the embedding of peptides in hybrid scaffolds can change the fiber surface increasing wettability and modifying the composition and structure of adsorbed serum protein layer, similarly to peptide coating, but, in addition, peptide embedding can modify the mechanical properties of the matrices because SAPs interact with both cristalline and amorphous PCL components [12].

The data collected in this study allowed us to correlate the peptide concentration in hybrid scaffolds with different matrix characteristics. In particular, we observed that the fiber diameter increased in line with the peptide concentration according to equations specific for each peptide. At the same, the wettability and percentage of nitrogen-containing functional groups of the surfaces increased with the increasing peptide concentrations in the hybrid matrices. The FT-IR analysis confirms that the electrospinning *per se* and the inclusion of SAPs in PCL do not alter the ß-sheet secondary structure of SAPs. This research showed the effect of different kinds of peptides and their concentrations (in the range of 2.5–15%) on the mechanical behavior of electrospun PCL-based meshes. Specifically, mechanical studies demonstrated that the increasing amount of RGD-EAK improved tensile modulus, maximum stress and maximum strain. Furthermore, an increase of modulus and maximum strain was attained by increasing GE3M concentration, whilst the enrichment with EAK and EAbuK produced particular variations. The biological data obtained by the evaluation of adhesion and differentiation of primary h-osteoblasts cultured on the different PCL-SAPs matrices are very interesting. Thus, even if the cells had similar physico-chemical properties in the different scaffolds tested, the hybrid matrices at specific peptide concentrations greatly improved the number of cultured osteoblasts as compared to the control PCL scaffolds. At the same, matrices enriched in non-SA peptide (i.e. 5% and 15% GE3M) did not negatively affect osteoblast vitality. These data could be explained by the increased wettability of the surface, different protein layer formation and the resulting easier interaction of cellular adhesion proteins with the surface of the scaffold. However, the performance of non-SA peptide-based matrices is quantitatively lower than SAPs. At the same time, both SA and non-SA peptides saw an increase in intracellular levels of Ca^2+^ compared to PCL. SA-based matrices, however, significantly increased the expression of mRNA transcripts involved in osteoblast differentiation whereas non-SA peptide did not. Indeed, the expression of IBSP, SPARC and Runx2 genes significantly increased in cells cultured on 5% EAbuK, 10% RGD-EAK, and 15% EAK. It is interesting to note that the same concentrations of these scaffolds also improved cell adhesion and calcium deposition in h-osteoblasts. Excluding the EAK-RGD matrix, the other scaffolds used in this work do not contain motifs (i.e., RGD) specifically recognized by cellular receptors. We hypothesize that in EAK, EAbuK, and GE3M the hydrophilicity of the surfaces mainly induces cell adhesion, whereas mechanical properties specifically observed in SAP-matrices but not in non-SAP-enriched scaffolds trigger the osteogenic-related gene expression profile. Indeed, the maximum strain of the matrices enriched with GE3M ranges from 0.20±0.01 mm/mm (2.5%) to 0.45±0.04 mm/mm (15%), whereas it spans from 0.04 to 0.17 mm/mm in SAPs. As a consequence, the matrices containing GE3M are more ductile than those enriched with SAPs probably because the SAP secondary structure (β-sheet) is stabilized by interlinked hydrogen bonds. Similar results were reported by Tsai S-W *et al*. [38] who described no difference between electrospun matrices of collagen or gelatin in terms of amino acid composition and hydrophilicity whereas the matrix stiffness, due to different secondary structure, influences the protein phosphorylation pattern and gene expression in MG63 osteoblast-like cells. Even if the ultimate tensile stresses of SAP-enriched PCL scaffolds account for the specific gene expression in our primary human osteoblast cells, there is no linear correlation between increasing SAP concentrations and the expression of osteoblast-related genes.

## Conclusions

An understanding of the mechanisms involved in the adhesion of cells to biomaterials improves not only the knowledge of the cellular processes but also allows us to identify new strategies in supporting tissue wound healing or, conversely, in preventing the anchorage of tumorigenic cells during cancer metastatic progression. In this important line of investigation, this study proposes the characterization of electrospun fibrous PCL scaffolds containing increasing concentrations of SAPs. So far, extensive international scientific literature on this field lacked systematic observations regarding the physicochemical/mechanical characteristics and biocompatibility of the electrospun fibers enriched with increasing concentrations of biomolecules. In this study, we showed that the physical and chemical properties of matrices such as fiber diameter, wettability and the amount of superficial peptides can be finely tuned by varying the concentrations of peptide in the electrospun solution. The current study also assessed the possibility to tailor the behaviour of the designed structures and, consequently, to find a compromise between mechanical and biological properties by suitably acting on materials chemistry, according to the specific application in the field of tissue engineering. The biological data confirmed that h-osteoblast phenotype is effectively sustained when cells are cultured on matrices enriched with SAPs rather than non-SAPs. These results were particularly evident when cells were cultured on matrices containing 10% RGD-EAK, 5% EAbuK, and 15% EAK. Based on the overall assessment of structural, mechanical and biological properties of the hybrid scaffolds, we proposed and motivated a correlation between h-osteoblast behavior and structural/mechanical characteristics of the matrices.

## Supporting Information

S1 Fig Normal plot of residuals. (TIF)

S2 Fig Parity plot of predicted and actual values for the response diameter. (TIF)

S3 Fig N1s signal evolution for the PCL-EAK scaffold. (TIF)

S4 Fig FT-IR spectra of the PCL-EAK scaffolds in the 1800–1500 cm^-1^ region at increasing peptide concentrations. The ordinate was normalized to the main C = O stretching band of PCL (ν_C = O_ 1740 cm^-1^), in order to evidence variations in the relative intensities of the other bands.
(TIF)

S5 Fig H-osteoblast vitality on PCL and PCL-SAP matrices. (TIF)

S1 Protocol Peptide synthesis. (DOCX)

S2 Protocol Cell culture and vitality assay. (DOCX)

S3 Protocol Calcium assay. (DOCX)

S1 Table Peptide and PCL quantities used to prepare the solutions for electrospinning. (DOCX)

S2 Table Considered Factors for design of experiments. (DOCX)

S3 Table Oligonucleotides and probes used in this study. (DOCX)

S4 Table Run experiments. (DOCX)

S5 Table Measured atomic ratios for PCL and PCL-SAP scaffolds and estimated peptide surface density. (DOCX)

S1 Text Estimation of surface peptide density trough two approaches. (DOCX)

S2 Text FT-IR analysis. (DOCX)
