# Supplementary figures and images for: Electrospun Scaffolds for Osteoblast Cells: Peptide-Induced Concentration-Dependent Improvements of Polycaprolactone
Source: PLoS One. 2015 Sep 11;10(9):e0137505. doi: 10.1371/journal.pone.0137505 (PMC4567138; doi:10.1371/journal.pone.0137505)

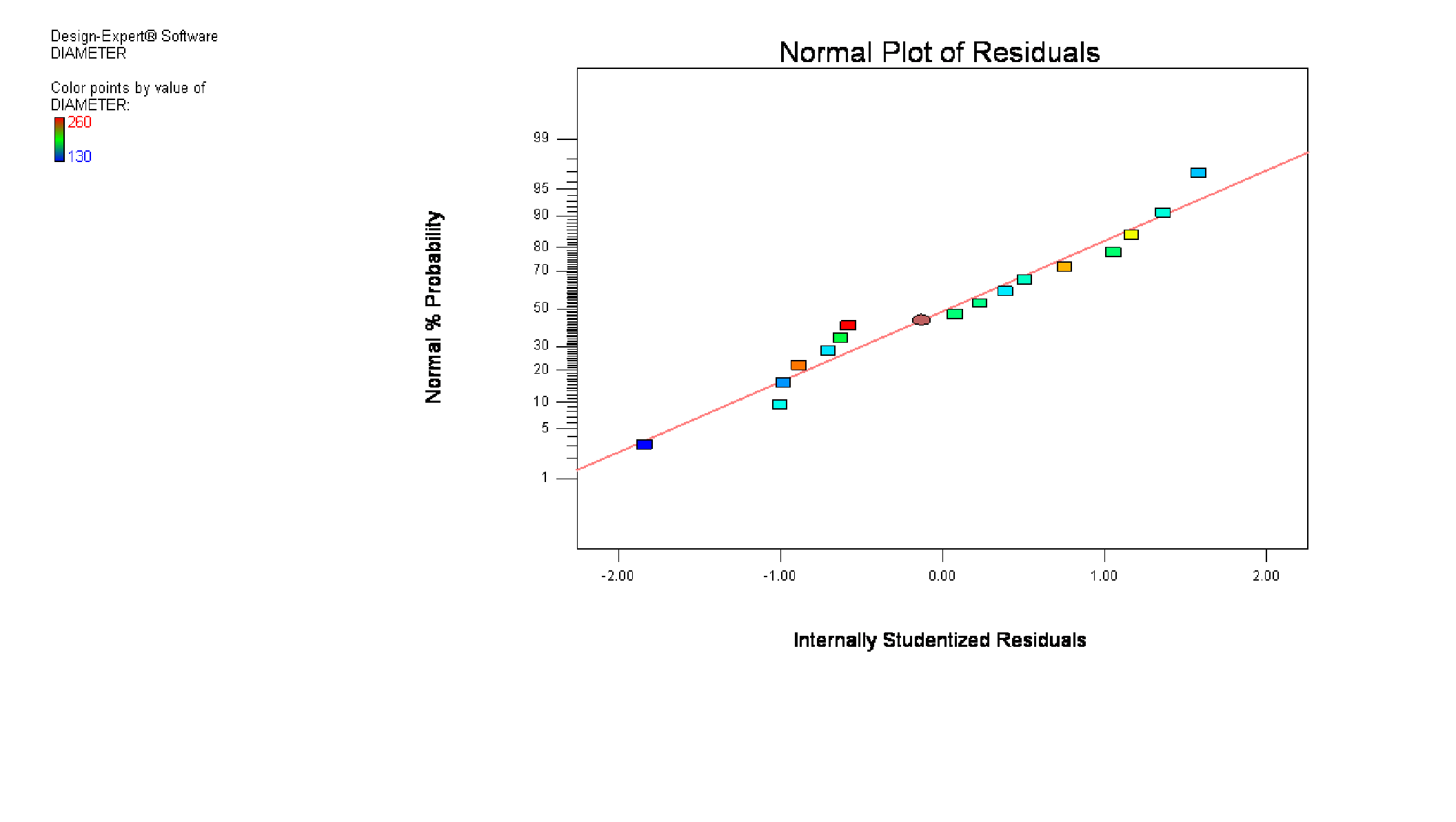

Supplement: S1 Fig — (TIF) [file pone.0137505.s001.tif]

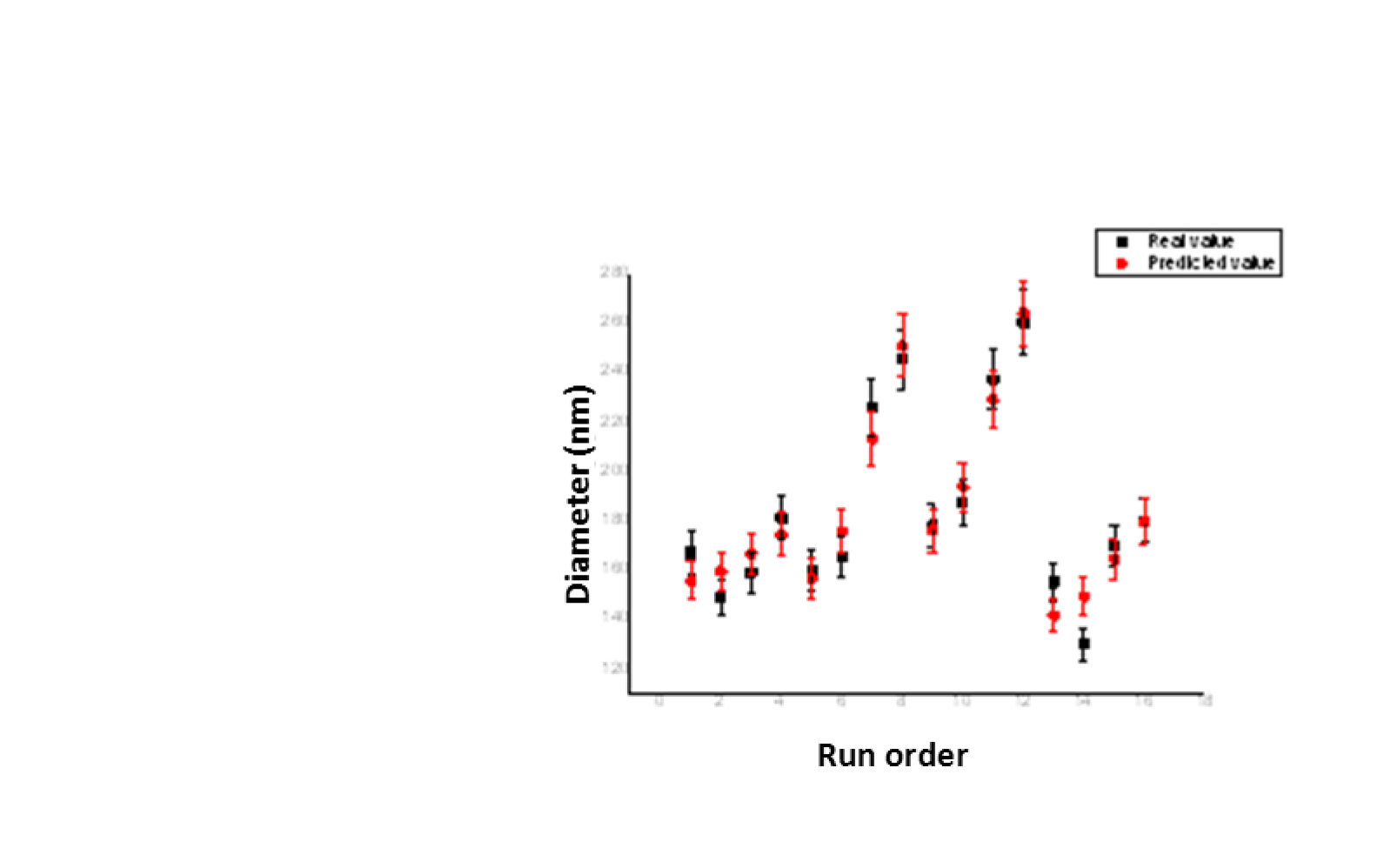

Supplement: S2 Fig — (TIF) [file pone.0137505.s002.tif]

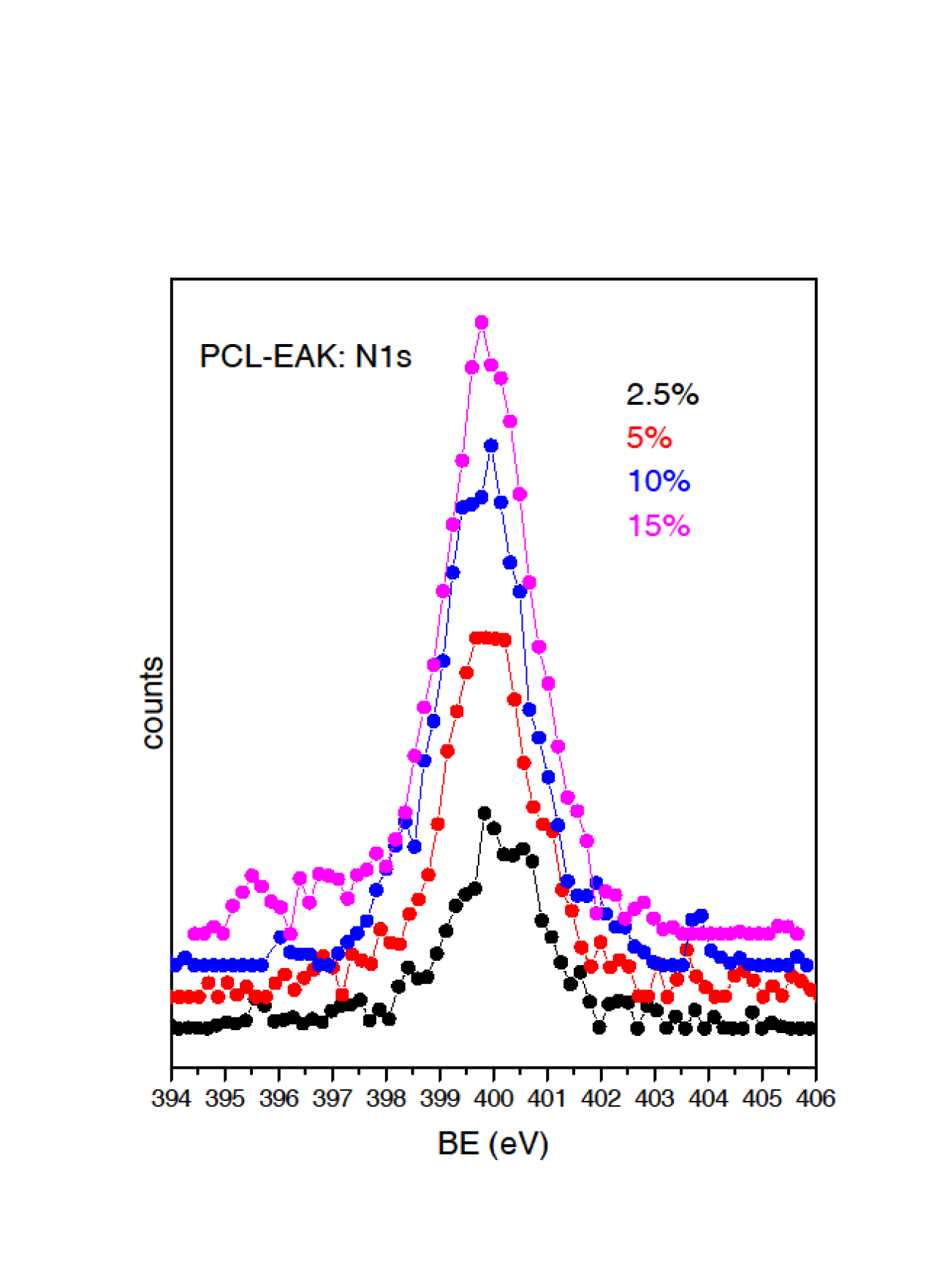

Supplement: S3 Fig — (TIF) [file pone.0137505.s003.tif]

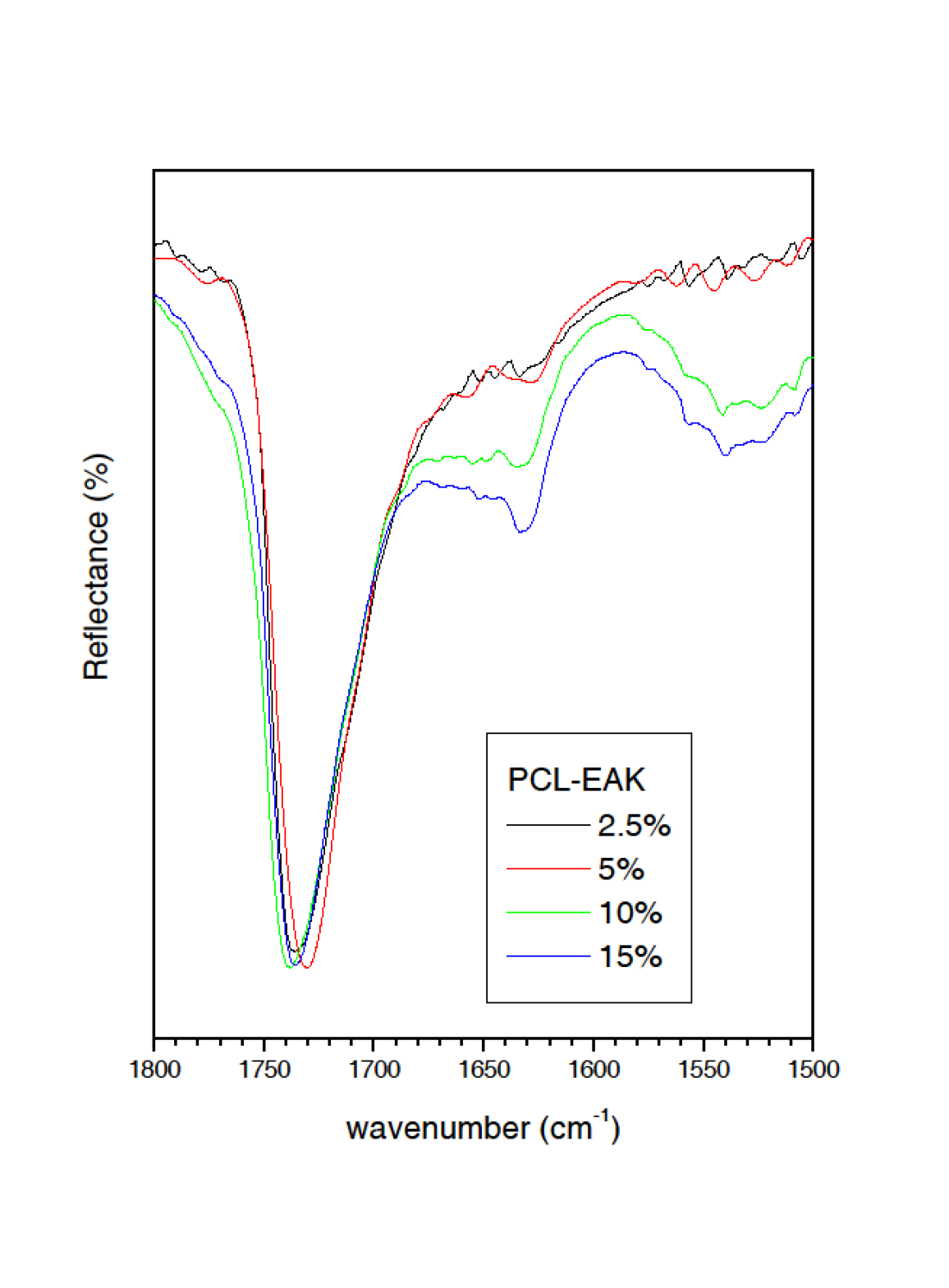

Supplement: S4 Fig — The ordinate was normalized to the main C = O stretching band of PCL (νC = O 1740 cm-1), in order to evidence variations in the relative intensities of the other bands. (TIF) [file pone.0137505.s004.tif]

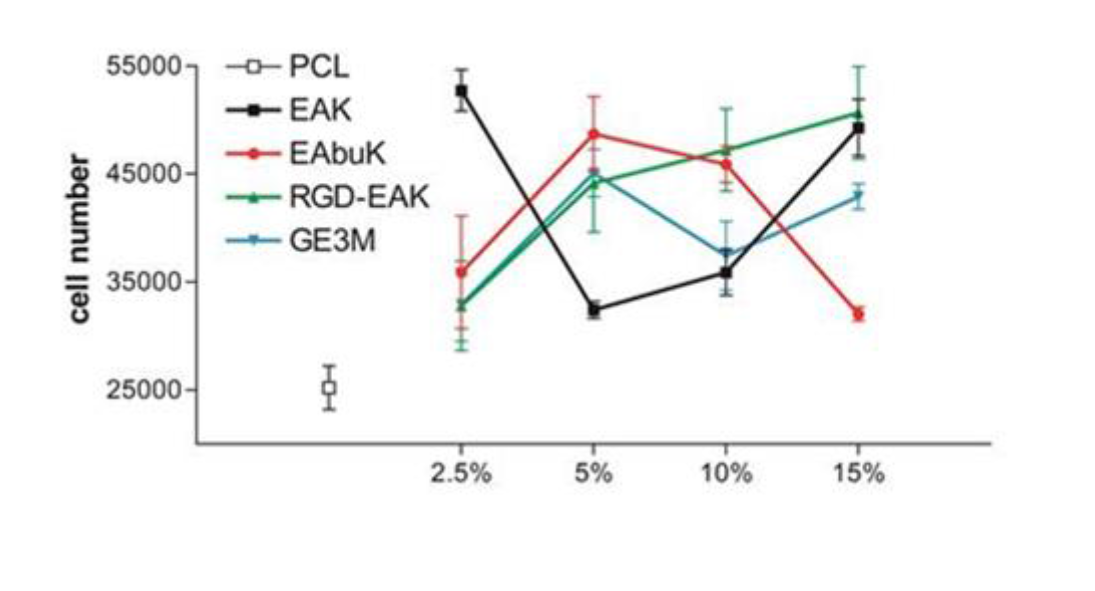

Supplement: S5 Fig — (TIF) [file pone.0137505.s005.tif]
