# Supplementary material for: Electrospun Scaffolds for Osteoblast Cells: Peptide-Induced Concentration-Dependent Improvements of Polycaprolactone
Source: PLoS One. 2015 Sep 11;10(9):e0137505. doi: 10.1371/journal.pone.0137505 (PMC4567138; doi:10.1371/journal.pone.0137505)
Supplement: S1 Protocol — (DOCX) [file pone.0137505.s006.docx]

## Peptide synthesis

*RGD-EAK*

This peptide present the adhesion motif Arg-Gly-Asp (RGD) condensed to the EAK sequence (Table 1). The peptide was synthesized on Rink-Amide MBHA resin (0.7 mmol/g) using Fmoc chemistry by Syro I synthesizer (Multisyntech, Witten, Germany). The following side-chain protections were used: *tert*-butyl ester (OtBu) for Asp and Glu; *tert*-butyloxycarbonyl (Boc) for Lys; 2,2,5,7,8-pentamethylchroman-6-sulfonyl (Pmc) for Arg. All condensations were carried out with double couplings (for each coupling: 5 equivalents of Fmoc-amino acid, 5 eq. HBTU, 5 eq. HOBt and 10 eq. DIEA for 45 min). The peptide was deblocked from the resin and deprotected from side chain protecting groups using 95% TFA, 2.5% TES, 2.5% (v:v:v) water mixture. Purification of the crude product was performed through reverse phase high performance liquid chromatography (RP-HPLC). The homogeneity (>99%) of the purified product was obtained by integration of the analytical HPLC peaks, whereas the identity of each product was ascertained by electrospray ionization time of flight (ESI-TOF) mass spectrometry (exp. mass = 1943.14 Da; theor. mass = 1943.16 Da).

*EAbuK*

The synthesis of EAbuK (Table 1) was carried out on 0.72 mmol/g Rink Amide MBHA resin using Fmoc chemistry by a Syro I synthesizer. The side chain protecting groups were: OtBu, Glu and Boc, Lys. The loading of the first amino acid was carried out with a double coupling. The following four insertions were carried out with single couplings (5 equivalents of Fmoc-amino acid, 5 eq. HBTU, 5 eq. HOBt and 10 eq. DIEA, for 45 min) and the remaining with double couplings. At the end of the synthesis the last inserted amino acid was Fmoc-deprotected. Crude peptides were detached from the resin and protecting groups were removed using a 95% TFA, 2.5% TES, 2.5% (v:v:v) water mixture. Purification of the crude product was performed through reverse phase high performance liquid chromatography (RP-HPLC). The homogeneity (>95%) of the purified product was obtained by integration of the analytical HPLC peaks, whereas the identity of each product was ascertained by ESI-TOF mass spectrometry (exp. mass = 1726.8 Da; theor. mass = 1727.05 Da).
