# Supplementary material for: Electrospun Scaffolds for Osteoblast Cells: Peptide-Induced Concentration-Dependent Improvements of Polycaprolactone
Source: PLoS One. 2015 Sep 11;10(9):e0137505. doi: 10.1371/journal.pone.0137505 (PMC4567138; doi:10.1371/journal.pone.0137505)
Supplement: S2 Protocol — (DOCX) [file pone.0137505.s007.docx]

**Biological Characterization**

*Cell culture*

Bone fragments were cultured in Dulbecco's modified Eagle's medium (DMEM)/Ham's F12 medium (1:1) supplemented with 20% fetal bovine serum (FBS), 1% sodium pyruvate, 1% non-essential amino acids, 1% antibiotic/antimycotic solution, 1 U/mL insulin (all from Gibco, Invitrogen, Milan, Italy).

*Vitality assay*

Cellular vitality was assessed by using the MTT (3-(4,5-dimethylthiazole-2-yl)-2,5-diphenyl tetrazoliumbromide) assay. H-osteoblasts seeded on electrospun matrices were incubated at 37°C for 2 h. At the end of the incubation, culture medium was discharged and non-adherent cells were removed by extensive washes in PBS. Electrospun samples were then incubated for 4 h at 37 °C with 100 μL of fresh complete medium containing MTT (5 mg/mL, Sigma). The reaction was stopped by adding sodiumdodecyl sulfate (SDS) solution 10% (w/v) acidified with 0.01 N HCl and samples were stirred for 12 h. To quantify the number of cells, a standard curve was obtained for each experiment by culturing a known number of h-osteoblasts in complete medium. At the end, cellular lysates (100 μL) were transferred to 96-well plates to determine the absorbance at 620 nm using a microplate reader (Sunrise, Tecan, Milan, Italy).
