# Supplementary material for: Electrospun Scaffolds for Osteoblast Cells: Peptide-Induced Concentration-Dependent Improvements of Polycaprolactone
Source: PLoS One. 2015 Sep 11;10(9):e0137505. doi: 10.1371/journal.pone.0137505 (PMC4567138; doi:10.1371/journal.pone.0137505)
Supplement: S3 Protocol — (DOCX) [file pone.0137505.s008.docx]

*Calcium assay*

In this study, h-osteoblasts were cultured on different electrospun matrices for 7 days. The complete culture medium was renewed every 2 days. At the end of the incubation time, samples were washed in PBS and added of 200 μL of trichloroacetic acid (TCA) 5% (w/v) in PBS. Plates were stirred for 30 min at 4°C. To measure the calcium content 100 µl of the cellular lysate were combined with HCl 3.6 mM, *o*-cresolphthaleincomplexone (100 μM, Sigma), and 2-amino-2-methyl-1-propanol (0.142 g/mL, Sigma) to a final volume of 300 μL, pH 10.7. A standard curve was obtained using dilutions (from 30 to 0 mg) of CaCO_3_. The absorbance of the purple-colored complex was determined at 620 nm using a microplate spectrophotometer (Sunrise, Tecan). To normalize the calcium levels to the cell density in the different scaffolds, protein concentration was determined in 25 μL of TCA-cell lysate using the bicinchoninic acid (BCA) method (Pierce Thermo Scientific, Rockford, IL, USA).
