# Supplementary material for: Electrospun Scaffolds for Osteoblast Cells: Peptide-Induced Concentration-Dependent Improvements of Polycaprolactone
Source: PLoS One. 2015 Sep 11;10(9):e0137505. doi: 10.1371/journal.pone.0137505 (PMC4567138; doi:10.1371/journal.pone.0137505)
Supplement: S1 Table — (DOCX) [file pone.0137505.s009.docx]

**S1 Table.** Peptide and PCL quantities used to prepare the solutions for electrospinning.

| Sample | Peptide | PCL |
| --- | --- | --- |
| PCL | --- | 71.5 mg |
|  |  |  |
| 2.5% EAK | 1.79 mg | 69.65 mg |
| 5% EAK | 3.57 mg | 67.36 mg |
| 10% EAK | 7.01 mg | 63.12 mg |
| 15% EAK | 10.60 mg | 60.03 mg |
|  |  |  |
| 2.5% EAbuK | 1.75 mg | 68.58 mg |
| 5% EAbuK | 3.60 mg | 68.74 mg |
| 10% EAbuK | 7.15 mg | 64.41 mg |
| 15% EAbuK | 10.79 mg | 61.14 mg |
|  |  |  |
| 2.5% RGD-EAK | 1.79 mg | 69.32 mg |
| 5% RGD-EAK | 3.57 mg | 67.84 mg |
| 10% RGD-EAK | 7.15 mg | 64.31 mg |
| 15% RGD-EAK | 10.72 mg | 60.73 mg |
|  |  |  |
| 2.5% GE3M | 1.79 mg | 69.74 mg |
| 5% GE3M | 3.59 mg | 68.10 mg |
| 10% GE3M | 7.11 mg | 63.94 mg |
| 15% GE3M | 10.75 mg | 60.91 mg |
