# Supplementary material for: Electrospun Scaffolds for Osteoblast Cells: Peptide-Induced Concentration-Dependent Improvements of Polycaprolactone
Source: PLoS One. 2015 Sep 11;10(9):e0137505. doi: 10.1371/journal.pone.0137505 (PMC4567138; doi:10.1371/journal.pone.0137505)
Supplement: S2 Table — (DOCX) [file pone.0137505.s010.docx]

**S2 Table.** Considered Factors for design of experiments

| Factor  Type | Name | Unit | Level I | Level II | Level III | Level IV | |
| --- | --- | --- | --- | --- | --- | --- | --- |
| A Numeric | Concentration | % | 2.5 | 5 | 10 | | 15 |
| BCategoric | Sequence | - | EAK | EAbuK | RGD-EAK | | GE3M |
