# Supplementary material for: Electrospun Scaffolds for Osteoblast Cells: Peptide-Induced Concentration-Dependent Improvements of Polycaprolactone
Source: PLoS One. 2015 Sep 11;10(9):e0137505. doi: 10.1371/journal.pone.0137505 (PMC4567138; doi:10.1371/journal.pone.0137505)
Supplement: S3 Table — (DOCX) [file pone.0137505.s011.docx]

**S3 Table.** Oligonucleotides and probes used in this study

| gene  [accession #] | amplicon  (bp^a^) | sequence | probe |
| --- | --- | --- | --- |
| GAPDH  [NM­_002046] | 66 | ^b^Fw: 5’-cgggaagcccatcacca-3’  ^c^Rv: 5’-ccggcctcaccccatt-3’ | 60 |
| IBSP  [NM_004967] | 61 | Fw: 5’-ttactaccaccaccagtgaagc-3’  Rv: 5’-gatgcaaagccagaatggat-3’ | 64 |
| SPARC  [NM_003118] | 81 | Fw: 5’-gaagagtttcacaaatccttcaaaat-3’  Rv: 5’-agactaagacacatgcaaatcacc-3’ | 8 |
| RUNX2  [NM_001024630] | 92 | Fw: 5’-cagtgacaccatgtcagcaa-3’  Rv: 5’-gctcacgtcgctcattttg-3’ | 41 |
| MKI67 [NM_002417] | 103 | Fw: 5'-tcaaggaactgattcaggagaag-3'  Rv: 5'-gtgcactgaagaacacatttcc-3' | 32 |

^a^bp: base pairs; ^b^Fw: forward; ^c^Rv: reverse
