# Supplementary material for: Electrospun Scaffolds for Osteoblast Cells: Peptide-Induced Concentration-Dependent Improvements of Polycaprolactone
Source: PLoS One. 2015 Sep 11;10(9):e0137505. doi: 10.1371/journal.pone.0137505 (PMC4567138; doi:10.1371/journal.pone.0137505)
Supplement: S4 Table — (DOCX) [file pone.0137505.s012.docx]

**S4 Table.** Run experiments

| Concentration  (%) | Sequence | Diameter  (nm) | St. Dev.  (nm) |
| --- | --- | --- | --- |
| 2.50 | EAK | 160 | 42 |
| 5.00 | EAK | 165 | 38 |
| 10.00 | EAK | 226 | 43 |
| 15.00 | EAK | 210 | 25 |
| 2.50 | EAbuK | 167 | 33 |
| 5.00 | EAbuK | 149 | 38 |
| 10.00 | EAbuK | 159 | 29 |
| 15.00 | EAbuK | 181 | 33 |
| 2.50 | RGDEAK | 155 | 38 |
| 5.00 | RGDEAK | 130 | 35 |
| 10.00 | RGDEAK | 170 | 32 |
| 15.00 | RGDEAK | 180 | 31 |
| 2.50 | GE3M | 178 | 32 |
| 5.00 | GE3M | 187 | 30 |
| 10.00 | GE3M | 237 | 40 |
| 15.00 | GE3M | 260 | 26 |
