# Supplementary material for: Electrospun Scaffolds for Osteoblast Cells: Peptide-Induced Concentration-Dependent Improvements of Polycaprolactone
Source: PLoS One. 2015 Sep 11;10(9):e0137505. doi: 10.1371/journal.pone.0137505 (PMC4567138; doi:10.1371/journal.pone.0137505)
Supplement: S5 Table — (DOCX) [file pone.0137505.s013.docx]

**S5 Table.** Measured Atomic ratios for PCL and PCL-SAP scaffolds and estimated peptide surface density

| Sample |  | C_2_/C_1_ | | C_3_/C_1_ | | O_2_/O_1_ | | O/C | | N/C | | N_pept_ | | C_pept_ | | (N/C)_pept_ | | d(%) | | n_pept_ | |  |
| --- | --- | --- | --- | --- | --- | --- | --- | --- | --- | --- | --- | --- | --- | --- | --- | --- | --- | --- | --- | --- | --- | --- |
| PCL | Meas. | | 0.27 | | 0.24 | | 0.89 | | 0.37 | |  | |  | |  | |  | |  | |  | |
|  | Calc. | | 0.25 | | 0.25 | | 1 | | 0.4 | |  | |  | |  | |  | |  | |  | |
| PCL-EAK | 2.5% | | 0.38 | | 0.28 | | 0.85 | | 0.30 | | 0.0065 | | 21 | | 68 | | 0.309 | | 2.1 | | 0.0019 | |
|  | 5% | | 0.38 | | 0.28 | | 0.79 | | 0.35 | | 0.013 | | 21 | | 68 | | 0.309 | | 4.2 | | 0.0039 | |
|  | 10% | | 0.40 | | 0.29 | | 0.71 | | 0.39 | | 0.030 | | 21 | | 68 | | 0.309 | | 9.7 | | 0.0095 | |
|  | 15% | | 0.38 | | 0.28 | | 0.62 | | 0.41 | | 0.037 | | 21 | | 68 | | 0.309 | | 12.0 | | 0.0120 | |
| PCL-EAbuK | 2.5% | | 0.37 | | 0.27 | | 0.87 | | 0.34 | | 0.0095 | | 21 | | 76 | | 0.276 | | 3.4 | | 0.0028 | |
|  | 5% | | 0.36 | | 0.26 | | 0.87 | | 0.30 | | 0.015 | | 21 | | 76 | | 0.276 | | 5.4 | | 0.0045 | |
|  | 10% | | 0.36 | | 0.26 | | 0.82 | | 0.34 | | 0.029 | | 21 | | 76 | | 0.276 | | 10.5 | | 0.0093 | |
|  | 15% | | 0.37 | | 0.27 | | 0.70 | | 0.38 | | 0.039 | | 21 | | 76 | | 0.276 | | 14.1 | | 0.0130 | |
| PCL-RGD-EAK | 2.5% | | 0.36 | | 0.26 | | 0.87 | | 0.355 | | 0.008 | | 27 | | 80 | | 0.337 | | 2.4 | | 0.0018 | |
|  | 5% | | 0.39 | | 0.27 | | 0.85 | | 0.375 | | 0.015 | | 27 | | 80 | | 0.337 | | 4.6 | | 0.0036 | |
|  | 10% | | 0.39 | | 0.28 | | 0.85 | | 0.36 | | 0.027 | | 27 | | 80 | | 0.337 | | 8.0 | | 0.0065 | |
|  | 15% | | 0.34 | | 0.26 | | 0.70 | | 0.41 | | 0.032 | | 27 | | 80 | | 0.337 | | 9.6 | | 0.0080 | |
| PCL-GE3M | 2.5% | | 0.38 | | 0.27 | | 0.82 | | 0.38 | | 0.0085 | | 16 | | 58 | | 0.276 | | 3.1 | | 0.0033 | |
|  | 5% | | 0.38 | | 0.26 | | 0.80 | | 0.40 | | 0.021 | | 16 | | 58 | | 0.276 | | 7.6 | | 0.0085 | |
|  | 10% | | 0.38 | | 0.27 | | 0.74 | | 0.38 | | 0.040 | | 16 | | 58 | | 0.276 | | 14.5 | | 0.0175 | |
|  | 15% | | 0.39 | | 0.26 | | 0.70 | | 0.40 | | 0.040 | | 16 | | 58 | | 0.276 | | 14.5 | | 0.0175 | |
