# Supplementary material for: Electrospun Scaffolds for Osteoblast Cells: Peptide-Induced Concentration-Dependent Improvements of Polycaprolactone
Source: PLoS One. 2015 Sep 11;10(9):e0137505. doi: 10.1371/journal.pone.0137505 (PMC4567138; doi:10.1371/journal.pone.0137505)
Supplement: S1 Text — (DOCX) [file pone.0137505.s014.docx]

Measured atomic ratios can be used to estimate the surface density of peptide molecules. A simple approach consists in estimating the peptide percentage from the ratio between the measured N/C ratio in the scaffold and the calculated N/C ratio in the peptide (N/C)_pept_, as follows: d (%) =[(N/C)/(N/C)_pept_]*100. Results obtained are summarized in columns 7-11 of S3 Table. There is a quite good agreement between the measured peptide surface density in the scaffold and the peptide concentration in the mother solution; the measured densities are slightly lower than expected for EAK 15% and RGD-EAK 10% and 15%, higher than expected for GE3M 10%.

A second approach consists in calculating the number of monomer unit of PCL per peptide molecule (n_PCL_); the reciprocal (n_pept_= 1/n_PCL_, last column of S3 Table) corresponds to the number of peptide molecules per monomer unit, yielding an estimation of the peptide surface density. In the measured N/C ratio, we must consider that all nitrogen atoms belong to peptide molecules, while carbons belong mainly to PCL and, to a lower extent, to peptide molecules. The measured N/C ratio can be therefore written as follows:

$\text{N/C}=\frac{\text{N}_{\text{pept}}}{\text{C}_{\text{pept}}+\text{C}_{\text{PCL}}}=\frac{\text{N}_{\text{pept}}}{\text{C}_{\text{pept}}+6\times\text{n}_{\text{PCL}}}=\frac{\text{N}_{\text{pept}}}{\text{C}_{\text{pept}}+6/\text{n}_{\text{pept}}}$🢡 $\text{n}_{\text{pept}}=\frac{6}{\frac{\text{N}_{\text{pept}}}{\text{N/C}}\text{-C}_{\text{pept}}}$

Where N_pept_ and C_pept_ (columns 7-8, S3 Table) are the number, respectively, of nitrogens and carbons in the peptide molecule, C_PCL_ is the number of PCL carbons per peptide molecule, 6 is the number of carbons in the PCL monomer unit and n_PCL_ is the number of monomer unit of PCL per peptide molecule. N/C ratios are experimental data (Fig. 5), while N_pept_andC_pept_ are calculated values. The equation can be used to calculate n_pept_= 1/n_PCL_ (last column of S3 Table).
