# Supplementary material for: Electrospun Scaffolds for Osteoblast Cells: Peptide-Induced Concentration-Dependent Improvements of Polycaprolactone
Source: PLoS One. 2015 Sep 11;10(9):e0137505. doi: 10.1371/journal.pone.0137505 (PMC4567138; doi:10.1371/journal.pone.0137505)
Supplement: S2 Text — (DOCX) [file pone.0137505.s015.docx]

FT-IR spectroscopy has been widely used to investigate the secondary structure of peptides and proteins; therefore, we have used it to check the conformation of the SAPs in our scaffolds. In the proteins and peptides IR spectra, the most diagnostic bands are those related to the peptide bonds, namely the amide I band, located between 1610 and 1690 cm^-1^, and basically related to peptide C=O stretching, and the amide II band, at about 1550 cm^-1^, related to N-H bending. The shape and position of the amide I band yields information on the peptide conformation. For β-helix and random coil structures the amide I band is found at about 1650 cm^-1^, for β-sheets between 1620 and 1640 cm^-1^, the lower the frequency the stronger the inter-chain hydrogen bond. For a parallel β-sheet conformation the amide I band is found at about 1635 cm^-1^, for antiparallel at 1615-1625 cm^-1^, due to stronger hydrogen bonds. Moreover, β-sheet structures show a second, less intense amide I band (amide I’) in the range 1670-1685 cm^-1^ _._ In the spectrum of our SAPS the amide I band (aI) is found at about 1620 cm^-1^, with an amide I’ band (aI’) at 1680 cm^-1^, suggesting an antiparallel β-sheet conformation; the amide II band (aII) is located at 1550 cm^-1^. S4 Fig. shows the spectrum in the 1800-1500 cm^-1^ range of the PCL-EAK scaffolds at increasing peptide concentrations; the ordinate was normalized to the main C=O stretching band of PCL (ν_C=O_ 1740 cm^-1^), in order to evidence variations in the relative intensities of the other bands. The spectra of the PCL-EAbuK and PCL-RGD-EAK scaffolds are perfectly alike. In the spectra of PCL-EAK scaffolds, the two features appear near the C=O stretching band of PCL, approximately in the position corresponding to the amide I and amide II peptide bands. The intensity of the new peptide-related bands increases with the peptide concentration in the mother solution. The amide I band is located approximately at the same wavenumber (1620-1630 cm^-1^) found for pure EAK and typical of β-sheet conformation, evidencing that the formation of the PCL-EAK scaffold does not affect the secondary structure of SAPs. The same results are obtained for PCL-EAbuK and PCL/RGD-EAK scaffolds. The amide I’ band is not visible because it is less intense and partially covered by the C=O stretching band of PCL.FT-IR investigation evidence that the native secondary structure of the SAP peptides is retained in the PCL-SAP scaffolds investigated.
